# Medico-Chirurgical Transactions. Second Series, Volume the Fourth

**Published:** 1840-01-01

**Authors:** 


### Medico-Chirurgical Review,

### No. LXIII.

**[No. 23 of a Decennial Series.]**

OCTOBER 1, 1839, TO JANUARY 1, 1840.

Medico Chirurgical Transactions. Published by the Royal Medical and Chirurgical Society of London. Second Series, Volume the Fourth. Octavo, pp. 344, Seven Plates, London, 1839.

The Volume before us contains twenty-three Communications, most of them possessed of considerable interest. They are as follows:

1. Cases of spasmodic disease accompanying affections of the pericardium; by Richard Bright, M.D. F.R S. physician extraordinary to the Queen, &c.—2, Case of malignant disease occupying one-half of the tongue, to which a ligature was applied from beneath the jaw; by James M. Arnott, surgeon to the Middlesex Hospital.—3. Memoir on tuphlo-enteritis; or inflammation and perforative ulceration of the cæcum, and of the appendix vermiformis cæci; by John Burne, M.D. physician to the Westminster Hospital, lecturer on the practice of medicine, &c.—4, Case of carditis; by Thomas Salter, Esq, F.R.S. of Poole.—5, Notices of the effects of lead upon the system; by James Alderson, M.A. and M.D. physician to the Hull General Infirmary.—6, Notices of the occurrences at the Small-pox Hospital, London, during the year 1838; by George Gregory, M.D. physician to the Small-pox Hospital; communicated by James M. Arnott, Esq.—7, Case of fracture of the coracoid process of the scapula, with partial dislocation of the humerus forwards, &c.; by John F. South, assistant-surgeon to St. Thomas's Hospital.—8, Statistical account of cholera in the Seamen's Hospital, in 1832; by George Budd, M.B. F.R.S. physician to the Seamen's Hospital.—9, Case of aneurismal tumour in the orbit, cured by tying the common carotid artery; by George Busk, surgeon to the Seamen's Hospital, Dreadnought.—10, On the softening of the coagulated fibrine; by George Gulliver, F.R.S. assistant-surgeon of the Royal Regiment of Horse Guards; communicated by Sir James M'Grigor, Bart.—11, Contributions to the pathology of the spinal cord; by William Budd, M.D., communicated by John G. Perry, Esq.—12, Memoirs on some principles of pathology in the nervous system; by Marshall Hall, M.D. F.R.S. L. and E. &c.—13, Case of enlargement from melanoid tumour of the prostate gland, in a child of five years of age; by R. A. Stafford, surgeon to the Marylebone Infirmary.—14, Case of congenital absence of the pericardium; by T. B. Curling, assistant-surgeon to the London Hospital, &c.—15, On a peculiar form of congenital tumour of the neck; by Cæsar Hawkins, surgeon to St. George's Hospital.—16, Cases of measles occurring oftener than once in the same individuals; by John Webster, M.D. consulting physician to the St. George's and St. James's Dispensary.—17, Remarkable case of dry gangrene occurring in a child three years and seven months old; by Samuel Solly, F.R.S. lecturer on anatomy and physiology, and lecturer on comparative anatomy, at St. Thomas's Hospital.—18, Statistical notices of one hundred and twenty cases of carcinoma uteri, with remarks; by J. C. W. Lever, Esq. communicated by Dr. Clendinning.—19, Case of a girl who voided from the urethra a number of entozootic worms, not hitherto described, with an account of the animals; by T. B. Curling, assistant-surgeon to the London Hospital. —20, Remarks on the acute form of anasarcous tumour of the scrotum; by R. Liston, surgeon to the North London Hospital.—21, An account of a fœtus of seven months, with its placenta partially adherent to a nævus occupying the scalp and dura mater; by Robert Lee, M.D. F.R.S. physician to the British Lying-in Hospital, and lecturer on midwifery at St. George's Hospital.—22, On the structure, physiology, and pathology of the persistent capsular investments and pulp of the tooth; by Alex. Nasmyth, M.R.C.S. —23, On the structure of the corpus luteum; by Robert Lee, MD. F.R.S., &c.

The bill of fare is not so bad. We shall select first the plain and practical dishes.

### I.—CASES OF SPASMODIC DISEASE, ACCOMPANYING AFFECTIONS OF THE PERICARDIUM. By RICHARD BRIGHT, M.D. F.R.S.

Dr. Bright relates three cases.

*Case* 1.—Dr. B. met Mr. Girdwood in consultation, and Dr. Sims on the 5th of April, 1836, on the case of a young man, seventeen years of age, who, about twelve days previously, had begun to complain of general rheumatic symptoms; pains in the limbs, with puffiness and swelling of the wrists, and some other joints, but the symptoms were not strictly those of acute inflammatory rheumatism.

"When all this was rather subsiding, about six days before I was called, peculiar spasmodic symptoms were said to have arisen, which had increased up to the time at which I first saw him, when I found him labouring under the most fully marked symptoms of severe chorea, except that the convulsion was more violent than is almost ever seen in chorea. His head was constantly thrown from one side of the bed to the other. His lips were closed, and opened with a smacking sound, and when desired to put out his tongue it was protruded with all the forced grimace and difficulty observed in chorea. The pulse was weak, and varied from 108 to 120, and there was an occasional sharpness followed by a feebleness and a variation in the beat, which induced us to pay attention to the action of the heart, but led to suspicions, rather than conviction, of that organ being more than functionally implicated in the disease; for we found in the excessive convulsion of the whole muscular system, sufficient to account for a great deal of the disturbance which there was in the circulation, and in the heart's action in particular.

For two or three days our remedies appeared to alleviate his symptoms, so that for a day or two it was not considered necessary to meet in consultation; but the relief was very temporary. The chorea became more severe, and the spasms put on the character of the most violent covulsions. There was likewise some wandering of mind, his apparent incoherence being however certainly increased by the convulsions and contortions of his mouth, which rendered his utterance difficult and his words indistinct. His condition after this became such that some degree of personal restraint was absolutely necessary, and I found that this had been adopted when I renewed my visit. The swellings at the wrist and on one hand again increased; he grew worse; and on the 15th, being about the sixteenth day after the first spasmodic symptoms, he died." 4.

*Dissection.*—The heart was found adhering to the free pericardium, by the most profuse effusion of firm semitransparent gelatinous fibrin. This was particularly the case about the base of the heart, while towards the apex the effusion was rather an opake serum. The substance of the heart was red, and the semilunar and mitral valves had each a fringe of vegetations, doubtless rather recent, forming a raised irregular line along the auricular side of the mitral, and along the aortal side of the semilunar valve. The lining membrane of the membranous portion of the auricle seemed to have a thin false membrane upon it. The edges of the lung which lay on the pericardium were slightly adherent to that membrane by inflammation. The brain and the abdominal viscera were most carefully and minutely examined, and all found perfectly healthy.

*Case* 2.—On the 1st of June, 1838, a gentleman, after exposure to cold, was seized with pain in the right side, and swelling, apparently rheumatic, of several of the joints. On the 5th, he sent for Mr. Balderson, who found him labouring under severe pain of the right side passing upwards to the shoulder, with a full pulse. He immediately bled him freely from the arm, and the blood was moderately buffed. The following morning venesection was repeated, on account of the continued difficulty of breathing, and the inability to lie down in bed; but the shortness of breathing seemed to increase, the pulse became quick and irregular, and often indistinct, and he complained towards the evening that he had a difficulty in swallowing. At eleven p. m. Dr. Bright saw him.

"This last symptom was so distressing, accompanied with so much difficulty in opening the mouth, that I was led to inquire very particularly, whether he had received any prick or wound which might be producing tetanus, spasm, or trismus, but I could discover nothing except a blow and cut received nearly six months before over his left eyebrow, but which had healed without difficulty, or subsequent ill consequences. I saw him swallow, although with much effort, and with a kind of convulsive catch. I found his heart running on very fast, and acting spasmodically, but could discover neither rubbing sound, nor any bruit. He had no cough, and at that time I detected no pneumonic crepitation in the lungs; the skin perspired very freely." 6.

It was concluded that rheumatic inflammation had affected the diaphragm, and probably the pericardium; and the prescription consisted of calomel, antimony and opium, mustard poultices to the chest, and purgatives.

"About two a. m. Mr. Balderson was called up to see him, on account of the rapid increase of the tetanic symptoms, and found that he had only been able to take one pill and one of the purgative draughts, and now his teeth were completely closed. At 9 a. m. I again saw him, and found him propped up in bed. The anxiety of countenance was excessive; the state of trismus was complete; so that it was impossible to get anything into his mouth, and his deglutition so completely interrupted that he was unable to swallow his saliva, and it was with the greatest difficulty he could get rid of it as it collected in his mouth. There were likewise some slight indications of opisthotonos and spasmodic action of the muscles of the back. His bowels had been twice relieved; his pulse was very rapid, often quite indistinct. We ascertained distinctly that there was some pleuritic effusion, with a rubbing sound, and likewise some pneumonic crepitation at the lowest part of the left lung." 7.

Blisters, mercurial ointment, opium suppositories, &c. were determined on, but by 2 p.m. there had been no relaxation of the spasm, and he had experienced two convulsive seizures. A third occurred during Dr. B.'s visit. It was quite epileptic in its character, and the countenance became purple and suffused—the eyes strained—the whole body convulsed. His pulse was very feeble, and his skin bathed in a most profuse perspiration. He was quite aware of his situation, though he rambled occasionally. He suffered three more distinct epileptic attacks, and died at 5 o'clock P.M. within twenty hours of the first appearance of the symptoms of dysphagia, on which the trismus and convulsions had rapidly followed.

*Dissection.*—There were traces of inflammation at the lower edge of the right and left lung. The lower half of the right pleura, as it covers the ribs and extends over the whole diaphragm, was highly inflamed, and covered with a thin coating of fibrin. The inflammation was, however, still more marked as it ran up towards the root of the lung and covered the right side of the pericardium, where the phrenic nerve was seen winding its way down the membrane in the midst of the most intense indications of inflammation, and as it ran over the diaphragm was covered with shreds of recent false membrane. The left pleura was also inflamed, and a chamber was formed at the lower part just where the diaphragm forms an agle, containing about half an ounce of straw-coloured serum intersected by bridles of recent lymph.

"The two cases," says our able author, "which I have now related are full of matter for interesting consideration, and in a practical point of view afford examples of the combination of inflammatory affections with convulsive symptoms, capable of masking each other in a manner which it is most important to bear in mind. In the former of these cases the inflammation of the pericardium was but darkly conjectured even to the last. In the latter case the inflammation of the pleura and diaphragm was much more early predicated, and yet the obscure story of a cut upon the forehead six months before had its weight on our minds in a case so exactly resembling traumatic trismus, and probably in some degree paralysed the desire for more large depletion; from which, however, I do not believe any benefit would have resulted, considering the complete relaxation of the pores of the skin, and the state of prostration to which the pulse was already reduced by the two previous bleedings. At the same time, when we find on examination the results of intense inflammatory action, it is not possible to feel confident that bleeding, even in the very advanced stages, might not have been serviceable. Had the state of the patient been such as to permit of the regular administration of medicine by the mouth, I have great confidence in the effect which calomel, antimony and opium would have produced; and this combination is, perhaps, a more appropriate remedy than large bleeding under such circumstances; but whatever the remedy we may feel inclined to adopt to overcome the disease, the great and important point is to impress upon our minds the fact, that the most violent attacks of spasmodic disease will occasionally owe their existence to inflammation of that portion of the pleura and the pericardium, where inflammation is often with difficulty detected—that part more particularly where the phrenic nerve in its course or its distribution is to be found." 10.

We would observe that the apparent prostration in such a case as the last does not seem to be an efficient reason against active depletory measures. These latter are no doubt useless or worse where actual disorganization has occurred in an important part. If there is a chance under such circumstances, it is from the reparative powers of the system, powers which violent measures would impair. But where the main lesion is a limited pleurisy, prostration must be rather the effect of the intensity of the inflammation on the system, and what relieves the inflammation must, consideratis considerandis, mitigate the prostration. All practical physicians will, however, allow, that cases of this description are much clearer after their issue than in their course, and perhaps there are few where the most observant men are more apt to be misled.

Dr. Bright, after quoting some opinions on the occasional connexion between chorea and rheumatism, observes that he has seen many cases of their combination and alternation, and some that convince him of the dependence now and then of the former on inflammation of the pericardium. We may mention the particulars of one.

*Case.*—Dr. B., about twelve years ago, saw a young lady, in consultation with Dr. Lister. The catamenia had been irregular, and she had suffered from rheumatic affections of the joints, though not in the genuine form of severe rheumatitis; several of the joints were puffy, slightly inflamed, aching and tender; and she had now begun to show symptoms of involuntary motion—the movements and actions, in fact, of complete chorea. The heart was agitated, and to it the chief uneasiness was referred; the pulse was hurried and irregular; on listening to the heart a "frottement" was distinctly heard. No doubt was entertained of the existence of an inflamed state of the pericardium. Remedies were applied, both local and general, under this impression, and the lady recovered completely, and is now living, the happy mother of a family.

And, after detailing several other cases, Dr. Bright adds:—

"Though I doubt not that in some cases the coverings of the cerebro-spinal mass may be, and are, implicated, yet I believe that the much more frequent cause of chorea, in conjunction with rheumatism, is the inflammation of the pericardium, and that the irritation is communicated thence, probably to the spine, just as the irritation of other parts, as of the bowels, the gums, or the uterus is communicated, and produces the same diseases; for I do not at all incline to the belief, that inflammation, in or about the spine, is necessary to induce chorea," 15.

The next case related by Dr. Bright is given with a very different view, with that, namely, of shewing, how symptoms may arise in chronic disease when the pericardium becomes implicated, which may give a peculiar aspect to the case, and embarrass the diagnosis.

*Case* 3.—A young lady, aged 17, three months prior to Dr. Bright's visit, had first begun to complain of a pain in the right foot and knee, and then in the groin, and extending up the side, so that it was supposed that the liver might be in fault, and slight mercurial action was induced; after this the pain became less, and a fulness in the iliac region and the groin seemed to diminish. Three weeks before Dr. B. saw her, some of the inguinal glands enlarged, and since that, some of the glands of the neck and under the left ankle; and for the last ten days a very hard swelling had taken place near the tuberosity of the ischium on the right side. It was quite plain that very extensive glandular disease was taking place; and although she had already lost one of her sisters of phthisis, as it was supposed, yet the general diffusion of the disease, without any pulmonary symptoms, seemed to render it probable that this was even more serious than simple tubercular disease. The country and sarsaparilla were prescribed.

" She was removed to Brixton, but I was again requested to see her on the 19th of June; all her symptoms having been increasing, and, in addition to the rest, she had become the subject of the most alarming attacks of dyspnœa, coming on generally in the middle of the night with agitation and shaking, and apparent convulsion of the diaphragm threatening death, and resembling an aggravated form of hysteric convulsion. I could now feel an abdominal hardness like a tuberculated omentum, and there were now to be discovered a string of small very hard glands, tender to the touch, running along the back on the left of the spine, near the lower dorsal vertebræ.

I saw her occasionally after this: the attacks of dyspnœa and convulsion returned frequently at night, and assisted in wearing her out gradually, and she sunk upon the 9th of July." 17.

*Dissection.*—The body, writes our author, was greatly emaciated. There were some subcutaneous tumors on the chest and abdomen. They were small, oval, flat, and some of them were softening at their centres.

Chest and lungs quite free from all tubercular deposit. The surface of the right lung had upon it several small, hard, malignant deposits, which, in one part, formed a fringe round the edge of the lung. The heart and pericardium formed a hard mass, firmly glued to the sternum by the white fungoid matter deposited in the anterior mediastinum, so that on raising the bone they were round like a hard tumor attached within.

The heart itself presented a curious specimen of disease: a thick layer of yellow malignant matter lined and covered the pericardium, both the portion attached to the heart and the reflected portion; the two deposits were strongly glued together in most parts, and were a quarter of an inch-in thickness. In other parts the two layers were easily torn from each other. The phrenic nerve was seen on the right side passing down, pressed upon, and almost embedded in this diseased mass.

The iliac, lumbar, and mesenteric glands were similarly diseased—as were the ovaries, and the broad ligaments.

Dr. Bright thinks there can be no doubt that the peculiar and very alarming train of symptoms which assisted in wearing out this patient, depended on the condition of the heart and pericardium, and the nature of the attacks would render it probable that the phrenic nerve was more particularly implicated in the irritation produced.

An interesting paper.

II. On a Peculiar Form of Congenital Tumour of the Neck. By Cæsar Hawkins, Surgeon to St. George's Hospital.

Mr. Hawkins, whose exertions to improve our acquaintance with the morbid growths are so well known and so successful, has communicated an instructive paper on this subject. After noticing the more familiar forms of encysted tumors, he proceeds to describe one of which he believes that no published account can be found. It is a form of congenital tumor, which is composed of many cysts joined together, in which the proportion of organized matter is so considerable, as to give a more solid character to the tumor, and make it deserve the name of cystic tumor, as much as the apparently analogous cases of cystic sarcoma, occasionally found in the breast, testis, or ovary of adults.

He presents two cases as fair samples of the affection.

*Case* 1.—A child about eight months old was taken to Mr. Hawkins by Mr. Julius, of Richmond, on account of a large tumor on the right side of the neck, which was of the size of a small orange at its birth, and had gradually increased since that time. The tumor now reached from the zygoma to the cricoid cartilage, and from the mastoid process to the chin; it projected outwards about three inches, so as to make the side of the face and neck appear nearly twice as large as the opposite side; and it extended also below the jaw, into the mouth, so as to push the tongue considerably to the opposite side and upwards. But although it caused much unsightliness, it appeared to occasion no pain nor inconvenience, and the child was quite healthy.

The skin was unaltered, and unattached to the tumor, and had much fat below it, and the outline of the tumor was smooth and uniform to the eye; on feeling it carefully, however, it was evident that there were several globular irregularities, some of which were of hard consistence, as if solid, and possibly glandular, but four others in the parotid and submaxillary glands appeared to contain fluid, which was the more probable, as two other cysts were perceptible below the tongue, transparent, like ranulæ, but containing a dark red fluid.

The cysts were punctured from time to time, as they refilled, and the same plan was pursued with several other cysts, which came forward as those first observed were obliterated; each cyst contained from a drachm to half an ounce of liquid, the contents of some being nearly clear water, with very little trace of mucus or albumen, but the secretion of others being of a darker colour, with more coagulable matter, so as to resemble melted currant jelly. Friction was also employed, with hydriodate of potash ointment in the intervals of the punctures.

This treatment was continued for about a year, when the disposition to secrete fluid appeared to be subdued, and the swelling was about a third of its original size, and what remained felt and looked like a loose pendulous bag of fat and skin, with two or three solid lumps, like glands, below. The child has been removed from Richmond for several years, but, when Mr. Julius last saw it, no tumor remained.

*Case* 2.—"The appearance of the tumour in this case was more complicated than in the next which I will mention, which Mr. Palmer attended, at the St. George's and St. James's Dispensary, and which he requested me to look at. The tumour was, in this instance, about the size of an orange, and was soft and elastic, moveable, and nearly pendulous, from its weight, and was situated in the same place as in the last case, in front of the ear, and below the jaw. It gave no pain nor inconvenience, and had scarcely increased in size since the birth of the child, who was now about a year old. The skin, as in the other case, was unattached, and had much fat below it, and there had been, I believe, some thoughts of removing the disease as a common adipose tumour. There were in it, however, several round bodies, two of which appeared to contain fluid, but the nature of the others was more doubtful. One of these, at the margin of the parotid gland, contained three drachms of clear liquid, but the other being punctured, no fluid escaped through the needle. With the final result of the case, I am not acquainted, as the child was not brought many times to the Dispensary."

The next case is brought forward by Mr. Hawkins, as eminently illustrative of the nature of these cases.

*Case* 3.—A child, eleven weeks old, was sent to Mr. Hawkins with a tumor in the neck. It was emaciated to the last degree, as, in fact, it had scarcely digested any food from its birth, almost every thing that was swallowed, or attempted to be swallowed, being nearly directly vomited again: it had also scarcely slept since its birth, as, whenever laid down in the horizontal position, it was instantly roused from slumber by impending suffocation; whatever rest it did obtain was therefore procured while held nearly upright in the arms. Notwithstanding this, however, it did not cry or breathe, when awake, as if there was any constant pressure about the glottis, nor was the tumor in the neck so tense as to account for the symptoms, unless some portion was intricately connected with the larynx and œsophagus, independently of the general tenseness of the whole tumor; which did not appear very probable, from the facility with which respiration was ordinarily performed. On the right side of the neck was a tumor, the prominent part of which was of the size of a large orange, and which reached from the zygoma nearly to the clavicle, and from the ear to the chin, across the upper part. It was soft and elastic, and the skin was healthy and unattached, and it seemed to be quite free from pain or tenderness.

Below the jaw, Mr. H. could detect three or four small cysts of fluid, and one or two smaller, and apparently solid bodies, like glands; but the greater part of the tumor in front of the ear did not fluctuate, nor present any irregularity of surface, but was soft and elastic, and compressible, like a subcutaneous nævus, which disease it resembled, also, in becoming more tense and prominent from crying or struggling, and below the mucous membrane of the mouth were several varicose vessels, like those observed sometimes in the neighbourhood of blood-vessel tumors.

The child died a few days afterwards, suffocated suddenly.

*Dissection.*—"When the thin skin covering the tumour was turned back, it appeared to be of the size of two oranges, separated by a deep sulcus, which was formed by the tendon of the digastricus muscle, which being much pushed forwards by the tumour, must have had its actions in connection with the larynx and pharynx much interfered with. The disease was now seen to be composed of a great number of small cysts, many hundreds probably, varying in size from a pea to a walnut, closely joined together, and composed of delicate membranes, like very fine peritoneum, but in some parts covered by fibrous structure, giving the cysts the appearance of thin pericardium, and very few of them were so far insulated as to admit of being dissected out without cutting through others. The fluid in most of the cysts was transparent, with scarcely any trace of coagulable matter, but in others the contents were of every shade of red, even as dark as venous blood, but without any coagulum, and evidently only coloured secretion.

The softness and elasticity of the prominent part of the tumour, arose from most of the cysts in that situation being flaccid, and only half filled; in other parts single cysts were so tense as to be quite unyielding, as if solid, and a feeling of solidity was also given by a few cysts being more closely joined together than those by their side; and in one or two parts a tense cyst projected into another in a flaccid state, so as also to feel like a solid body; although the only really solid bodies were two or three small absorbent glands mixed with the cysts. The portion of the tumour in front of the ear was covered by a thin layer of condensed parotid gland, another part of which, in a healthy state, lay behind the cysts, through the middle of which ran the portio dura, and external carotid artery. The submaxillary gland was pushed out by other cysts, so as to be loose below the skin, and all the vessels and nerves at the base of the jaw were surrounded by some of the cysts, and curiously twisted in their course.

On dissecting deeper, the cysts were found to extend along the front of the spine, behind the pharynx and œsophagus, some being as high as the basilar process, and some as low as the sixth cervical vertebra; and along the whole length of the neck the cysts surrounded the carotid artery, jugular vein, and nervus vagus, which were even separated from one another by some cysts formed in their sheath. None of these bodies were very closely united to the œsophagus and pharynx, and there seemed to be nothing morbid about the glottis, except a slight thickening of the mucous membrane." 239.

Mr. Hawkins makes some remarks on diagnosis:—

"It is evident from this account, that the numerous cysts which compose the tumour are formed in the common cellular tissue; each separate cyst, it is to be presumed, being like the single serous encysted tumour met with in many parts of the body, at every age; why such numbers should be formed at once before birth, does not appear, unless it arise from the peculiarly lax and watery nature of the cellular membrane in the fœtus, especially, perhaps, in the neck, where I believe such a tumour to be more common than elsewhere.

The number of cysts existing in the tumour, and the different degrees of consistence of the whole or of separate parts in different cases, arising, as the dissection demonstrates, from the size and state of tension, and relative position of individual cysts, make the diagnosis of the tumour somewhat obscure. When numerous and full of liquid, and small in size, they feel like enlarged glands, or other solid globular bodies; when numerous and only half filled, they become soft and compressible; but in both these cases the existence of fluid is difficult to be detected in comparison with those cases in which only a few larger cysts exists.

The resemblance to fatty tumours is considerable, and the deception is assisted by the quantity of fat generally situated beneath the skin, and filling up the inequalities of the surface of the tumour. They are also much like a subcutaneous nævus, which is so often developed in the same situation, when the cysts are half-filled; in the fatal case I have related, the softness and compressibility of part of the tumour, its increase from exertion, and the varicose vessels within the cheek and mouth, made me think it probable that some part of the tumour was composed of blood-vessels, though the nature of the remainder was clearly perceptible. In each case that I have seen, however, the existence of globular bodies in some of which fluid was perceptible, distinguished the tumour from every other kind likely to be formed in infancy." 41.

We naturally inquire what treatment may seem most likely to succeed.

Excision may appear easy but would probably be difficult if not impossible. We may refer to the dissection already given, and we may refer also, to a case of Mr. Arnott's in which that gentleman operated. The tumour, indeed, was situated in a more favourable spot, behind the sterno-mastoid muscle. A single cyst was opened first, when the infant was a month old, and repeated once more, and when five months old this was laid open in order to remove what appeared to be a solid body, but which was found to be composed of a multitude of small cysts, as in the cases previously narrated. The tumor was followed under the sterno-mastoid muscle and carotid artery, and behind the pharynx, and its total excision being found impossible, a ligature was tied round the deeper part. The child ultimately did well, but the ligature was not entirely thrown off for three months, during which time repeated attacks of erysipelas occurred.

The results of Mr. Hawkins' observations on the subject of treatment may be thus set forth :—

1. The cysts may be emptied, from time to time, by a grooved needle, so that there is no scar whatever; or by a lancet, when they are situated in the mouth; the punctures heal directly, and the emptying the larger cysts appears to assist the action of other remedies upon the cysts themselves.

2. Pressure may be employed, especially after the evacuation of the fluid, in some situations, as in front of the ear, although, of course, this means is generally inapplicable, on account of its obvious interference with respiration, mastication, and deglutition.

3. Stimulant applications may be constantly employed, of such a strength as to excite moderate inflammation, but stopping short of suppuration, in order to avoid deformity; to avoid which Mr. Hawkins has directed their intermission for a short time after the punctures.

"The cysts do not appear, however, much disposed to inflammation, beyond the degree necessary for their obliteration and absorption, after the fluid has been got rid of. The applications I have made use of have been the ointment of hydriodate of potassa, rubbed on by the hand; a solution of a drachm of iodine and two scruples of hydriodate of potassa in an ounce of water, painted over the tumour by means of a camel's hair brush; a lotion, of half an ounce of Goulard with two ounces of spirit, and six of camphor mixture, applied by means of folded linen; or one composed of from one to two drachms of muriate of ammonia, mixed with two ounces of vinegar and spirits of wine, with eight or ten of water." 243.

We have found the tincture of iodine, repeatedly applied with a brush, a good application, where such local means are wanted. It produces continued desquamation of the cuticle, and does not occasion permanent discoloration.

Mr. Hawkins observes that this method of treatment must needs be tedious, hence he has seldom seen cases to the end.

His readers must feel obliged to Mr. Hawkins for this useful communication.

III. Notices of the Effects of Lead upon the System. By James Alderson, M.A. and M.D. Physician to the Hull General Infirmary, &c.

Dr. Alderson has seen two cases in which paralysis of the nerves of vision resulted from exposure to the influence of lead. Acting on the hint afforded by the use of splints for paralysis of the extensor muscles of the fore-arm, Dr. Alderson excluded light from the paralysed eyes in these cases, and with the happiest effect.

*Case* 1.—Dec. 27, 1833.—Elizabeth Clayton, aged 25 years, single woman, was admitted into the Hull General Infirmary. States that she has worked for seven or eight years in the lead works, and has never before been attacked with paralysis. She first perceived the approach of paralysis about six weeks ago. The hands were the first to lose their power; after about three weeks her eyesight began to be defective. The sight is at this time completely lost. The pupils are somewhat dilated. Her legs are also affected with paralysis, but she scarcely knows how or when they became affected. She states that she has done all sorts of work in the lead works, but is of opinion that what is called "picking," is the most deleterious. The girls of the works are employed on this duty every other week only.

Dr. Aldersou gives us, in a note, the following account of "picking."

When lead, in its blue metallic form, is first placed in the earthen pots, it is rolled up or cast in forms, and placed over vinegar; it then remains for some time, (six or eight weeks,) embedded in manure and bark, which keeps it at a proper temperature for the action of the vapour of the vinegar upon metal. When first opened out for work or separation, there arises a heated vapour, which is thought by those employed in the works to be very pernicious. The pots are then taken out of the beds and carried to a table, to be "picked over." The lead is then unrolled, or passed between rollers and crushed, to force off the encrusted carbonate, or white lead of commerce. That which is white is separated from the metallic part, and it is this separation, or "picking," as it is called, which is found to be so pernicious. During the mechanical separation, by crushing, the finer portions of the carbonate will be suspended in the atmosphere, and breathed by the girls whilst employed in the work.

The following medicines were ordered for the patient. A drachm of sulphate of magnesia, with two minims of laudanum, in an ounce of the compound infusion of roses, every four hours. Ten grains of the compound ipecacuanha powder at bed-time, and a stimulating liniment to the neck and spine, and a generous diet.

She was in the house three weeks on this plan, without any improvement in the vision, though during this time her other paralytic symptoms were perceptibly improving. With a view to take off the stimulus of light from the nerves of vision, a bandage was then directed to be applied over the eyes, so as to exclude the light altogether. The bandage to be worn day and night.

By the 18th of February she could read large print and was made an out-patient, soon after which she was discharged from the hospital perfectly cured.

*Case* 2.—On June 10, 1835, Elizabeth Dyson, aged 18 years, was admitted into the Hull General Infirmary, suffering from complete lost of sight, with paralysis of the hands. States that she has worked nine months in the lead works, and has had colic several times; that castor oil was always sufficient in her attacks of colic to remove the complaint.

A bandage, as before, was ordered over the eyes, and to be kept strictly applied, so as to exclude all light; and the same plan of treatment was adopted as in the last case.

June 24th. Some power of vision returning; better in her other paralytic symptoms.

July 8th. Eye-sight quite restored; ordered quinine.

July 15th. Discharged from the hospital cured.

We add the following remarks.

"I may be allowed to add, that several cases of paralysis, without loss of vision, and numerous cases of colic, have also within the last nine years fallen under my care, both in the hospital and out of it. Those of paralysis, without colic, I have found, for the most part, hitherto to yield to the compound infusion of roses, with salts and laudanum. Whilst those of acute colic always give way to a steady perseverance in drastic purgatives, assisted by purgative injections.

In the milder cases, the old remedy, calomel, colocynth, and opium, in repeated doses, answers admirably; whilst in the more severe cases, croton oil frequently repeated, until relief be obtained from the bowels, is more efficacious than any other remedy with which I am acquainted. The form and dose which in my experience is found to answer best, is two drops of the oil, with ten grains of magnesia, a drachm of mucilage, and an ounce of cinnamon water, every two or three hours.

I willingly bear testimony to the excellence of this remedy in cases which resist the milder and more common treatment, in overcoming what was formerly supposed to be spasm in the bowel; but which we now believe, reasoning analogically from those parts which we have the means of observing, to arise from paralysis of portions of the intestine.

This state of the intestine, in the more severe cases, requires the most powerful drastics in order to induce the bowel to empty itself of its contents. It may admit of doubt in what way the effect is produced by drastics, whether as a stimulus to the paralysed part of the intestine, or to the more healthy part of it, by which so much increased action is induced, as to carry the contents of the bowel through and beyond the paralysed part. Certain it is, that the most powerful drastics are required, and are well borne by the patient." 87.

Dr. Alderson adds:—

*Some Cases of Paralysis from the unsuspected absorption of Lead, in consequence of drinking rain water, kept in lead cisterns.*

On the 18th July, 1837, Dr. A. saw, in consultation with Mr. Craven, Mr. Thackray, aged 63.

He was labouring under paralysis of the upper extremities, and partial paralysis of the lower. The hands, with the palms turned inwards, hung by his sides, and rather forward, perfectly powerless, rather swollen, and of a livid hue. He had no power of moving the fore-arm, nor of rising from his chair without assistance. When he walked to his bed-room, he required the assistance of a servant on each side of him, and then his knees bent under him, and his gait was tottering. His voice was a good deal altered, and the control over the tones of the voice much impaired. His countenance was blanched, but not sallow; the functions of the brain, as an organ of intellect, not interfered with. He complained of great pain in the lower part of the body, accompanied by costiveness, and of pain across the legs and arms. He would frequently shed tears from slight causes.

"I learnt from Mr. Craven, that his bowels acted only every three or four days, and then only in consequence of having taken active medicine; that in a former attendance, eighteen months previously to my being called to attend him, Mr. Thackray, had an epilectic seizure. During the fit he fell out of bed, and dislocated the left shoulder, since which period he has not been affected with epilepsy.

The fact, that the sister of Mr. Thackray's late wife, who lived in the house with him, was last year attacked with paralytic symptoms, precisely similar to those of her brother-in-law, excited my attention, as a remarkable coincidence. These had supervened upon a debilitated constitution, which had for some years shown signs of giving way. She died paralytic, without any cause of the paralytic symptoms having been made out.

As there was no relationship by blood between them, it appeared to me more than probable, that some common agent existed as the cause of the symptoms in both.

On inquiry, it was ascertained that the water used by the family, and which was supposed by them to be more pure than any supplied to the town, was rain-water, collected from the top of the house by means of lead gutters, and received into a cistern lined with lead. It was filtered for use, and had a sweetish taste, which had often been remarked by the servants. On testing the water in conjunction with Mr. Robert Craven, agreeably to the recommendation of Dr. A. T. Thompson, we obtained distinct evidence of the presence of lead.

It was now deemed advisable by us to call in to our assistance Mr. Pearsall, a most able chemist, to determine the precise condition in which the lead existed, and the proportion contained in the water.

On examining the sides of the cistern, in conjunction with Mr. Pearsall, and scraping them with the nail, the white carbonate of lead was abundantly manifest, especially in the angles of the cistern, where the soldering had, probably by galvanic action, assisted and expedited the change. Efflorescence was here distinctly to be seen.

It was now pretty evident that the source of the paralytic symptoms was lead in the water; and, in consequence, Mr. Thackray was immediately put on a similar plan to the cases which I have already detailed, viz. compound infusion of roses, with suphate of magnesia and laudanum, and a generous diet allowed, with stimulus in moderation. Subsequently quinine, strychnine, frictions, and electricity.

I am happy to have it in my power to say, that Mr. Thackray is now able to walk almost any distance, to use his arms pretty well, though he has not regained, and probably never will regain, complete power over the wrists and fingers.

He is now able to enjoy life, which was previously becoming a burden to him; and he is very grateful for the providential discovery of lead in the water used by the family, and which is now entirely discarded for culinary purposes.

Many of the usual symptoms were manifested in the servants, but in a minor degree; they have been, more or less, tormented with colicky pains, and by constipation; but not having resided for any long period subjected to the influence of the water, it was not to be expected that they would be affected with the more chronic symptoms, as paralsyis, &c.

It is worthy of notice, that it was always remarked that when Mrs. Martin (Mr. Thackray's sister-in-law) went on a visit into Lincolnshire to her native air, she recovered wonderfully from her paralytic symptoms, and came home much invigorated and restored; but on her return to Hull, the symptoms of paralysis soon returned. The action of lead on the system was not at that time suspected.

This fact accords, however, with our observations on the general action of this poison in the system in those whose pursuits and avocations lead them to handle it or to be amongst it. In painters we witness an aggravation of symptoms on a return to their accustomed employment; even the wearing of unclean apparel has been supposed to re-induce the disease in those who have recently suffered from it." 91.

### IV. Case of Malignant Disease occupying one-half of the Tongue, in which a Ligature was applied from beneath the Jaw.

Mr. Arnott observes that when malignant disease of the tongue has extended to its basis, the application of a ligature from the *mouth* has been found impracticable, and the case abandoned as hopeless. But some operations performed by M. Jules Cloquet and M. Mirault have tended to modify this feeling of despair.

*Case* 1.—In a man who had disease on one side of the tongue, and which could not be removed by incision or the ligature applied through the mouth, M. Cloquet operated thus:—Having made a small incision in the mesial line of the neck above the os hyoides, he passed a curved needle, having an eye at the point and fixed in a handle, through the base of the tongue, and carrying the handle backwards caused the point to project between the teeth. Through the eye two ligatures were passed, and the needle being withdrawn carried the ligatures through the tongue. The free part of this organ anterior to the frenum was next divided in its middle, and the needle being reintroduced at the wound in the neck, its point was made to appear in the floor of the mouth just before the frenum. The oral ends of both ligatures were next passed through its eye, and the needle being withdrawn, one of the loops thus formed was disposed longitudinally on the middle of the tongue and in the angle of its division; the other across, so as to surround it on the outer side. Being tied, the cancerous part was included between them. The operation terminated unfavourably, the patient dying in three days.

*Case* 2.—" In a case of alleged cancerous tumours," continues our author, " with ulceration of the tongue, which M. Mirault met with in a woman, (aged 23,) and which occupied the centre of the organ in almost its entire width, two modes of operating presented themselves to the mind of that gentleman, who was not at the time aware of M. Cloquet's case. One of these was to apply two ligatures to the tongue immediately in front of the arch of the palate, in order to intercept the circulation through the part, and to divide it transversely, and then, ' dans un second temps,' to extirpate the anterior portion of tongue, by detaching it with scissars from the floor of the mouth.—The other was to tie both lingual arteries, and having thus guarded against hæmorrhage to extirpate the cancer. M. Mirault determined upon adopting the latter proceeding, but in attempting it, he failed in finding the left lingual artery, though be succeeded in tying the right. Fifteen days subsequently, in order to control the circulation through the left, he placed a ligature around this half of the tongue, for which purpose he made an incision from about a finger's breadth under the chin to the os hyoides, and by this a needle was carried through the base of the organ into the mouth, out of which he drew the needle: this was again carried into the mouth, and through its floor, directly in front of the arch of the palate on the left side of the tongue, and brought out at the wound under the chin.

The ligature being tightened cut its way through the tongue in nine days, and during this time the tumour sensibly diminished, and the edge of the ulcer became less hard and prominent. To complete the operation nothing now remained but to detach the parts from the floor of the mouth, and to divide the right half of the tongue.

M. Mirault preferred doing this 'en deux temps,' and reversing the order just mentioned, he resolved to effect the division of the right half by means of a ligature, as had already been effected on the left.

Having done so, the beneficial results were so remarkable, that the remaining step of the operation was postponed—then abandoned—in fact, the tumour rapidly decreased, and the ulcer began to heal, so that in twenty-seven days from the last operation, the former was reduced to the size of a nut, and the latter was in great part healed. Two months subsequently the tongue had resumed its natural size, and presented a very dense cicatrix on its surface, but the tumour had entirely disappeared.

At this time the patient could move the tongue freely, could speak so as to be readily understood, and the sense of taste was retained on both sides of the tongue." 32.

*Case* 3.—Hannah Hayward, fifteen years of age, was admitted into the Middlesex Hospital, on the 8th of May, 1838: the right half of the tongue was occupied by a tumour which projected from its upper and under surface, extended from nearly half an inch of the apex to the isthmus faucium, protruded at the edge between the teeth, and in the mesial aspect overstepped this line, and by compression had considerably reduced the width of the left or sound half of the organ. The bulk and form, generally, of the swelling, were those of a pullet's egg, but with irregular outline. The prevailing colour was purple, in parts, however, yellowish grey, apparently from effusion of lymph underneath the cuticular covering. Posteriorly, the upper surface was covered by warty excrescences: anteriorly, it presented a vesicular appearance, from serous effusion into the papillary structure. The substance of the tumour was firm, solid, and unyielding to compression. Soreness of the part was complained of, and pain was felt in the ear. Trifling bleeding had occasionally taken place from the part, and was readily excited by rough handling, but soon ceased. There was no enlargement of the neighbouring lymphatic glands, and the patient looked well. She had never menstruated.

The disease had begun as a small blue swelling nine years ago, but increased rapidly six weeks previously, after some pills which she had taken for fits. It is unimportant mentioning the remedies employed, as they were useless, and the disease advanced.

"The size of the swelling had now materially increased, filling the concavity of the palate, and the sub-lingual space; it measured 2½ inches in length,—1¼ in breadth,—and 1½ in thickness,—reducing the left half of the tongue to a narrow strip. In the ordinary situation of the parts, the limits of the disease could not be seen; but by causing the tongue to be protruded, and drawing it forcibly over to the left side, the boundaries of the tumour could be traced by the finger passed along its outer edge, under the arch of the palate, where sound structure could be felt beyond. On the mesial aspect and superior surface the warty excrescences previously mentioned, had so overlapped the left half of the organ as to render it impossible to trace the sulcus naturally existing in this situation. The presence of these growths, and more especially the prominence in this direction of the swelling itself, rendered it more difficult to distinguish the boundaries of the disease on the upper surface posteriorly, which were, however satisfactorily made out. Inferiorly, in the corresponding situation, this, of course, from the attachment of the parts, could not be done, but from the examination, as far as it could be carried, and the degree of persistent mobility of the organ, it was inferred that the disease did not include the whole of the basis below." 23.

*Operation.*—This was performed on the 6th of June. The able operator thus narrates it :—

The patient being seated, the head slightly extended, and the os hyoides felt, an incision was made from over it upwards and forwards, an inch and a half in length in the mesial line, through the skin, cellular substance, and raphe, of the mylohyoid muscles. With the edge of the knife, but chiefly by its handle, way was made for the finger between the two genio hyoid and the two genio glossi muscles. A tenaculum was next passed through the apex of the tongue, by means of which it was drawn out of the mouth, and held so during the subsequent part of the operation by Mr. Mayo, (Mr. Tuson, Dr. Warren of Boston, U.S., and Mr. B. Phillips, being likewise present). Into the wound in the neck, a strong needle with an eye at the point in a fixed handle, was now conducted and passed through the basis of the tongue into the pharynx, a little to the left of the mesial line: the loop of ligature which it carried was then, by means of a blunt hook, drawn forwards out of the mouth, and the needle withdrawn from the wound over one of the ends. The loop being cut, two ligatures were obtained; one of these was placed along the upper surface of the tongue, so as to bound the disease on its left side, and carried through the apex of the tongue from above downwards by means of a large curved needle, through which the oral end of the other ligature was *now* also passed. Fixed in a porte-aiguille, this needle was next carried through the floor of the mouth immediately behind the last molar tooth, on the right side, directed at first, and for the greater part of its course, perpendicularly downwards, then inclined mesial, and brought out at the incision in the neck. There were thus two ligatures, the four ends of which hung out of this wound: one of the loops was so disposed as to encircle the right half of the tongue at its basis beyond the tumour; the other was placed longitudinally on the upper surface of the tongue, longitudinally and obliquely below. Being tied, (and this was done as tightly as possible,) the diseased mass was circumscribed posteriorly, laterally, and, in some measure, inferiorly. A third ligature was now passed through the fore-part of the tongue, so as to isolate at this point the diseased from the healthy structure.

The immediate effect of the operation was, that the tongue became fixed in the mouth, and the patient unable to articulate or swallow. For a fortnight all food and medicine were conveyed into the stomach through an elastic gum catheter. We need not mention details of little moment.

On the eleventh day the condition of the parts was as follows: the *diseased* was completely separated from the *sound* half of the tongue by a deep trench, so as to give it a truly bifid character, and the trench was continued across the basis, seeming to extend through the whole thickness of the part. The tumor was lessened in size by the sloughing of its surface,—it had wholly lost its vesicular and warty character, was of a pale red colour,—felt firm and resisting generally, and hard posteriorly. About the middle of its dental edge its colour was livid, and close to this on the upper surface the latter was irregular and ulcerated. Reunion by granulation had commenced between the diseased and sound portions of the tongue, but this was easily broken down by the probe. It was now evident that the former part derived some vascular supply from below, and the following method was employed to cut this off.—A loop of silver wire, properly bent, was passed over it, from the mouth, carried and depressed into the trench already mentioned as surrounding it, and being drawn forwards, the diseased part was found to be placed completely above the level of the loop. The two ends of the wire were next passed through a double polypus canula, and this being carried home under the tumor, to what may now be considered as its neck, the ligatures were tightened, the death of the part effected, separation ensuing on the fifth day (the seventeenth from the first operation.) The patient left the hospital on the 10th of July, and there is yet no appearance of a recurrence of the disease. Mr. Arnott adds:—

"It will be recollected that the mere tip of the tongue on the right side not having been included in the disease was not removed. For some time the patient was much annoyed by her inability to distinguish the taste of substances coming into contact with this portion of the apex, and by its getting perpetually pinched between the teeth. Although the former condition remains unchanged, as I have this day (Nov. 13th) ascertained by the application of sapid bodies, she has become reconciled to it, and the latter does not now occur. For a longer time she was incommoded by food getting into the cavity left by the operation, and either remaining there unnoticed, or when she was aware of it, requiring the employment of the finger for its removal. But partly from the space there being diminished, owing to the development of the previously compressed parts, and partly from having acquired a greater degree of power in the use of the tongue towards its displacement, she is not subjected to as much inconvenience as heretofore.

The motions of the tongue are restrained by the cicatrix attaching it to the bottom of the mouth, and the tip cannot be protruded beyond the teeth, but a stranger would not discover by her speech that she had lost so large a portion of it, he would merely remark that she lisped." 29.

A case well deserving of perusal, and very creditable to Mr. Arnott.

### V. Notices of the Occurrences at the Small-pox Hospital, London, during the Year 1838. By George Gregory, M.D. Physician to the Small-pox Hospital.

This is a short, but a valuable report. We cannot much curtail it.

The total number of patients admitted into the Small-pox Hospital during the year 1838 was 712—416 males, and 296 females.

Of them 17 had diseases not variolous (such as lichen, spurious measles, &c.)

Total, having small-pox in some of its forms, 695.

Of them there died 187, being at the rate of 27 per centum.

Of these 695 there had been vaccinated in early life 302, of whom 30 died, being at the rate of 10 per cent.

The remainder, 393, were unvaccinated, and of them 157 died, being at the rate of 40 per centum.

No patient applied for admission having previously been *inoculated!*

In 1781 the admissions were 646; of them 257 died, being in the ratio of 40 per cent., the exact proportion in which the unvaccinated died during the year just passed.

Had the like rate of mortality extended to all the patients admitted, the total deaths would have been 277.

Dr. Gregory observes :—

"90 lives therefore were saved in 1838, more than in 1781; and I think there can be no question that, while some portion of this may be attributed to the improved state of the present Hospital, compared to that in Cold-bath Fields, which received casual patients in 1781, the largest share is due to *vaccination.*

Even at this time the arrangements at the Hospital are wholly inadequate for the numbers. In the months of March and April typhus fever broke out in the wards in consequence of overcrowding, proving fatal to 12 persons, of whom 6 had been vaccinated, and 6 had not.

This would reduce the deaths by small-pox after vaccination to 24, being in the ratio of 8 per cent.

Of the 302 patients received with small-pox after prior vaccination, 97 had the disorder in that extremely mild form, commonly called *'varicelloid.'*

The remainder (205) had it in variable degrees of severity, from the most distinct mild, through the several grades of semiconfluent modified and semi-confluent unmodified, to the confluent modified, simple confluent, and confluent malignant.

Great difficulties were necessarily experienced in determining who had been really vaccinated, of those who assumed to have undergone that process. The cicatrix was our chief guide; but this often failed us, from the swollen and pock-covered condition of the arm at the time of the patient's admission.

The same difficulty occurred occasionally with those who professed never to have undergone vaccination. The highly modified aspect of the disease in many of them afforded strong presumption of prior vaccination, which the appearances of the arm gave some countenance to.

The errors on the one side may fairly be taken as balancing those on the other." 97.

The following statements have an important bearing :—

The average age at which the patients were received, both the vaccinated and the wholly unprotected, was 21.

79 children were received *under ten years of age*, unvaccinated, of whom 31 died; 5 only at the same age, vaccinated, of whom none died.

The earliest age at which any *vaccinated* person was received was eight years.

The earliest age at which death took place after prior vaccination was fifteen years.

Between ten and twenty years of age, 246 *unvaccinated* were received, of whom 90 died.

Between twenty and forty years of age, 181 *unvaccinated* were received, of whom 86, or nearly one-half, died.

The increased severity of the disease, as life advances, is proved in like manner to hold true in respect to the vaccinated. Between fifteen and twenty-nine years of age (inclusive) 92 vaccinated persons were admitted, of whom only 6 died. (Say 6 per cent.)

Between twenty and thirty (inclusive) 163 vaccinated persons were admitted, of whom 23 died, being 15 per centum.

The great bulk of the vaccinated cases were between the ages of eighteen and twenty-four.

13 were received having small-pox after vaccination, between the ages of thirty-one and thirty-five, of whom one died.

4 were received above thirty-five years of age, vaccinated, all of whom survived.

The greater number of the vaccinated underwent that process in early infancy, and but a few at a period of life when they could remember the event.

By far the larger proportion were vaccinated in the country, and exhibited only *one cicatrix* on the arm.

We subjoin a short table, with the expression of a wish that our experienced friend, Dr. Gregory, will not be costive of his communications.

*Return of the Ages of the several Persons admitted into the Small-pox Hospital in* 1838.

| Ages of Patients having Small-pox. | UNPROTECTED. | | VACCINATED. | |
|---|---|---|---|---|
| | Admitted. | Died. | Admitted. | Died. |
| Under 5 years .......... | 42 | 20 | 0 | 0 |
| From 5 to 9 .... ....... | 37 | 11 | 5 | 0 |
| From 10 to 14 .......... | 30 | 8 | 25 | 0 |
| From 15 to 19 .......... | 103 | 32 | 92 | 6 |
| From 20 to 24 .......... | 113 | 50 | 108 | 15 |
| From 25 to 30 .......... | 45 | 23 | 55 | 8 |
| From 31 to 35 .......... | 12 | 7 | 13 | 1 |
| Above 35 years.......... | 11 | 6 | 4 | 0 |
| Total .......... | 393 | 157 | 302 | 30* |

* Of these 6 died of superadded typhus.

### VI. Remarks on the Acute Form of Anasarcous Tumour of the Scrotum. By R. Liston, Esq. Surgeon to the North London Hospital.

Mr. Liston thus introduces six cases :—

"I propose to bring under the notice of the Society, briefly, a few cases of a different character from those above alluded to; cases of rapid distention of the scrotum with serosity, in which destruction of the cellular tissue and skin can, on the contrary, be arrested only by very early and free incisions.

This distention is or is not attended by redness or erythema of the surface; but there is reason to think, from the suddenness of the accession, and from the appearances on exposing the cellular tissue, that there is no actual inflammation of its texture; there being no induration, nor any appearance of lymph or puriform fluid in the areolæ.

The affection has generally supervened upon abscess or ulcer, perhaps trifling, in the perinæum or groin. Its accession has been sudden, the swelling and tension becoming very great and alarming even within a few hours. The most dependent part, generally the posterior, will be found at a veiy early period to present one or more deeply-seated ash or tawny-coloured spots; these extend; the integument is speedily involved; and, unless active measures be adopted, the entire coverings and investments of the testicles will be destroyed, and these organs exposed.

The progress of the case will depend materially upon the state of the patient's system, and upon the nature of the infiltration. Cases of œdematous swelling of these parts, the result of injury or of irritation in the neighbourhood, are continually occurring, in which none of these dreadful consequences arise, or need be feared. But in hospital practice, in some of those in the working classes, perhaps badly fed and clothed, or cachectic from any cause, and during unhealthy seasons, whilst erysipelas is prevalent, the surgeon will do well to look with suspicion at any sudden swelling in this region, and be prepared to treat it actively.

The fluid effused, and which falls, as it were, into the cellular tissue of the scrotum, is often dark, putrescent, and acrid; it causes destruction of all the parts it comes in contact with, and subsequently of the skin, as certainly, and sometimes as rapidly, as if the parts were infiltrated by urine. Cases of sloughing of the coverings of the genital organs are very generally supposed to arise only from this latter cause; and it is with a view of directing attention to the diagnosis, that the following cases are brought before the Society.

The same treatment, it is true, is so far required in either case. The scrotum must be freely opened at the point, where, in consequence of the pressure being greatest, the destructive process has commenced: but of course, in the acute anasarcous tumour, or as it may, in some cases, very properly be called, inflammatory œdema, there can be no occasion for interference with the urinary passage, by the application of instruments, either from without or within.

The history of the case, and the absence of a hard tumour in the perinæum, which attends upon and very generally precedes infiltration of urine, will enable the surgeon to form a correct opinion of the nature of the mischief, and to direct his procedures properly and satisfactorily." 291.

We hesitate to coincide entirely with Mr. Liston in the view he takes of its nature. We believe it to be diffuse inflammation of the cellular tissue of the scrotum, analogous to diffuse inflammation of the cellular tissue elsewhere. It is very true that the redness of the skin is not usually marked, though we never saw an erythematous suffusion absent—and that lymph and pus are not present if incisions are made early. But these circumstances may admit of explanation by the structure of the part and the habit of body which is usually present. This being commonly cachectic, the inflammation is of a low kind, and characterized by an abundant effusion of serum, and little vascularity. If incisions are not practised, we certainly have seen both lymph and pus beneath the skin, with sloughs of cellular tissue.

The effusion taking place into the spongy texture of the dartos, and into the loose sub-dartoic cellular membrane, unprovided with fat, and merely traversed by vessels, accounts for the rapidity and extent of the effusion. In a certain low condition of the bodily powers, inflammatory action coming upon such a part, serum is likely to be suddenly poured out into it, and the structure of that part, as well as the weakness of those powers, are little calculated to throw up a barrier of lymph, or to form pus with readiness.

In cases of stricture and of fistula perinei, it is often difficult to determine whether a sudden swelling of the scrotum depends on this affection, or on effusion of urine. No doubt, many cases of reputed effusion of urine from stricture, are really of this description. Fortunately, the treatment is much the same in both, early incisions constituting the pith of it.

Mr. Liston's cases rather prove, we think, our position. We will take a couple at random.

*Case* 1.—"J. A., aged twenty-three, a sailor. Admitted into the Edinburgh Royal Infirmary, March, 1834.

He had been shipwrecked on the coast of England, and exposed to the inclemency of the weather for many hours. He afterwards travelled on foot, towards the north, in great distress, scantily clothed, and badly fed. An abscess had formed near the verge of the anus, for which he applied for relief at the hospital. The opening of the abscess, which was anterior to the orifice of the bowel, and superficial, was somewhat enlarged. Two days afterwards the scrotum was found much swelled : the swelling had come on suddenly during the night. At the visit at twelve, A.M., there was a slight erythema of the perinæum, and the scrotum also was reddish, shining, and much distended, with a grey appearance at the lower and back part. Free incisions were made on each side of the septum, and slight sloughing of the skin and cellular tissue took place.

The after treatment consisted in the exhibition of antimonials, followed by bark, and the patient made a rapid recovery." 292.

Here after a slight operation, the scrotum swells suddenly, and is described as "reddish, shining, and distended," while the cellular membrane subsequently sloughs. Such circumstances would assuredly be deemed indicative of diffuse cellular inflammation elsewhere.

*Case* 2 (V.)—"S. H., a labourer, of spare habit. Admitted into the North London Hospital, May 19th, 1835.

The disease for the relief of which he applied to the hospital, was an abscess over the knee-joint, which was cured ; but, just before he was about to be discharged, he complained, for the first time, of slight pain and swelling in the groin: and, on examination, a small fistulous opening was discovered, surrounded by a blush of inflammation. He then said that the opening had existed for some time. The next day (May 28th) rigors took place; the skin was hot; tongue furred; bowels confined; and the erysipelatous redness extending over the groin and upper part of the right thigh, passing downwards to the scrotum.

On the 29th the scrotum had become very much swollen, and was attended with discolouration at its most dependent part. The cellular tissue of the prepuce was also much infiltrated.

A free incision was made in the lower part of the scrotum, near the raphé, and the cellular tissue found in a state of gangrene. A small vessel was divided at the lower part of the incision, from which about ℥xv. of blood were allowed to escape. Fomentations were applied to the groin, poultices to the scrotum, and twelve leeches to the epigastrium, as he complained of slight tenderness in that region. Warm-water dressing was the next day applied to the wound; and, under this treatment, joined with purgatives, antimonials, and tonics, he recovered, and was discharged from the hospital on the 14th of June." 296.

Here the case is still more distinct. Erysipelatous inflammation spreads to the scrotum, and *then* comes on the peculiar affection of *that*. What can be more evident than the modification of inflammatory action by the special structure of the part? Erysipelas of the face when it invades the lids is particularly liable to occasion infiltration or abscess, or sloughing, because the cellular tissue of the lid is devoid of fat. Erysipelatous or diffuse inflammation, when it invades the scrotum, is particularly liable to occasion infiltration and sloughing there for a very similar reason. The lid is small and in many respects more favourably circumstanced than the scrotum, which influences the results.

Such is the view (it may be an erroneous one) which we are inclined to take of this affection. We conceive that the term diffuse inflammation of the scrotum would be preferable to "acute anasarca," which we cannot help thinking less applicable.

But whatever notion may be formed of the minor matter of name and nosological character, Mr. Liston equally merits our thanks for bringing the subject before the profession. Things are often known in a small circle and not known to the public. Whoever diffuses information deserves well.

### VII. A CASE OF ANEURYSMAL TUMOUR IN THE ORBIT, CURED BY TYING THE COMMON CAROTID ARTERY. By GEORGE BUSK, Surgeon to the Seaman's Hospital, Dreadnought.

Mr. Busk has partially adopted the Horatian precept, and kept this case by him for three years, that time might sanction the expectations of success. It were well if all case reporters acted similarly.

*Case.*—A healthy seaman, aged 20, was admitted into the Seaman's Hospital, July 13, 1835, with severe concussion of the brain, following a blow on the right side of the head. A variety of symptoms followed, which we do not think it necessary to particularise, suffice it to say that there ensued onyx of the left eye, ulceration of the lower half of the cornea, small-pox, and great noises in the head.

About the latter end of January, the ulcer on the cornea was filled up, leaving an opaque cicatrix which occupied more than its lower half. Through the upper half, which had retained its transparency, he could see very well when the upper eyelid was raised. The globe of the eye was very prominent, and the eyelids were generally swollen; several large, tortuous conjunctival vessels were observed, passing to the lower edge of the cornea. On the 1st of February, when examining the eye, Mr. Busk, for the first time, perceived a distinct pulsation of the globe; and on more close investigation, detected also a firm pulsating tumor in the upper and inner part of the orbit, immediately within the superciliary ridge, and about half an inch in its transverse and longest diameter.

This tumor appeared to be situated between the levator of the eyelid and the bone, and was not evident externally; but when the eyelid was raised it caused some projection of the loose conjunctiva. The pulsation of the tumor was accompanied by a very distinct thrill, which could also be felt on pressing the parts in its immediate neighbourhood. Through a small ivory stethoscope a very loud whizzing sound was communicated; this could also be heard on applying the instrument over the inner canthus of the right eye, and on the left side of the frontal bone, as high as the roots of the hair, and nearly as far back as the ear. The eye felt hot and uneasy; but, otherwise, he had no pain, and complained principally of very loud noises in the head: those in the right ear like the sound of church bells, and those in the left, the breaking of waves on the sea-shore.

Mr. Busk adds:—

"As temporary pressure on the left common carotid put a stop to the pulsation in the tumour and eye, and to the accompanying sounds, as well as to the noises in the head, it appeared clear that they all depended on a common cause, and that, probably, an aneurism of some vessel within, or close upon the orbit. I was not at the time aware of the two similar cases to which I shall afterwards refer; but entertained no doubt as to the propriety of treating this like an aneurism in any other part, and of tying the left common carotid. Twenty ounces of blood were taken from his arm the same evening; and, on the following day, February 2, the carotid was tied in the usual place and manner. Immediately on tightening the ligature, the pulsation of the tumor, and that communicated to the globe, ceased, together with all the sounds, both internal and external. In the evening, four hours after the operation, obscure pulsation could be felt in the tumour, which had not however resumed its original size. The whizzing sound could also be plainly heard through the stethoscope, and over the same extent of surface as before the operation. The internal noises were also, at intervals, nearly as loud as before, but were sometimes absent. There was no pulsation in the temporal artery. He complained of pain in swallowing. Pulse 110. An opiate was given, and an evaporating lotion applied to the face and head.

The next morning the pulsation was very obscure, and the sounds much diminished. He had not slept, and complained much of pain on deglutition and on coughing, and of severe pain in the left hypochondrium. Pulse 120, sharp. A bleeding of sixteen ounces afforded immediate relief, and in the evening he was easier in every respect. On the 4th no remains of the tumour could be felt, and all pulsation was gone from the orbit, nor could any sound be heard by means of the stethoscope. The internal noises were also quite absent, and his hearing was somewhat improved. Pulse 100 soft; skin moist; and tongue clean.

On the 6th the eye appeared less vascular than before the operation, and was not so prominent. He had occasionally some noise in the left ear, but none in the right. A small quantity of grumous blood passed from the left nostril during the night. On the 11th the eye was still more retracted within the orbit, and there was no pain nor pulsation. He sat up several hours. On the 15th the ligature came away, and on the 18th his state is thus noted:—The wound was much contracted; the eye hardly prominent and much less vascular. The ulcer in the lower half of the cornea filled up, leaving a dense leucoma, to which ran a large tortuous vessel. Vision perfect as far as the loss of transparency of the cornea would allow. There was no pulsation, nor any noises. Pulse 70; soft; appetite good; no pulsation in the temporal artery. The paralysis of the left side of the face and muscles of the globe continued complete; but sensation was perfect except on the left side of the nose; and he was more deaf in the left ear than in the right. From this time no material change took place, up to the time of his discharge from the hospital, on the 28th March 1836. He complained almost constantly of pain, commencing about the infra-orbitary foramen, and extending towards the nose, which was in some degree relieved by the use of veratria." 130.

He returned to his occupation as a seaman, made a voyage to Quebec, no trace of tumor or pulsation could be detected in or about the orbit, and no pulsation could be felt in the carotid or any of its branches.

After alluding to several cases more or less similar, Mr. Busk, in a note, mentions one which occurred to Mr. Scott, at the London Hospital, of a parallel character to this.

*Case.*—A boy, William Cooper, who had fallen into a ship's hold, was brought to the London Hospital, October 2nd, 1834, with concussion of the brain, violent contusion and swelling on the right side of the head, and protrusion of the right eye, which was fixed and motionless, the pupil dilated and vision lost. There were no symptoms of cerebral pressure.

He gradually recovered from the concussion of the brain, the eye becoming gradually more prominent. The protrusion of the globe immediately after the accident, without symptoms of cerebral compression, proved that it arose from extravasation of blood within the orbit, and the further continued protrusion rendered it probable that the aperture in the vessel from which the blood escaped had not closed:—the symptoms were not so acute as to indicate suppuration. As the globe became more prominent, pulsation was felt on pressure, and when the palpebræ were separated, the eye could be distinctly seen to be propelled forward at each stroke of the heart.

Pressure was made on the globe; but, after being borne for two days, it occasioned so much pain that it was abandoned. On Tuesday, November 10, just after examining the eye, a profuse arterial hemorrhage occurred from the nose. Mr. Scott commanded it by pressure on the common carotid artery, and immediately secured that vessel. The protrusion of the globe directly receded in a great degree, and its prominence afterwards gradually diminished.

Mr. Busk is inclined to believe that these are instances of true aneurysm, rather than of erectile tumors, and for these reasons:—

"1. The sudden accession of the disease attended with pain.

2. Its rapid increase.

3. The powerful pulsation occurring in the tumours when recent and small; in all of which circumstances do they differ from the erectile tumours as described by Mr. John Bell under the name of aneurism by anastomosis; and when to this is super-added the strong aneurismal whizzing sound, which was heard over so extended a space in the present instance, and which may possibly have existed in the others, as they were attended with similar internal noises, the presumption of the affection being in all really an aneurism of some vessel in the orbit, appears to me materially strengthened; and Mr. Guthrie's case may possibly lead to the supposition of the ophthalmic artery itself being the seat of the affection.

The success of the treatment by ligature of the carotid cannot be adduced as further proof of the correctness of this view, since the same success attended that operation for an undoubtedly erectile tumour of the antrum in the hands of Mr. Pattison; for an external nævus in one of these cases in which it was employed by Mr. Wardrop." 134.

### VIII. Statistical Notices of One Hundred and Twenty Cases of Carcinoma Uteri, with Remarks. By J. C. W. Lever, Esq.

Mr. Lever makes some observations on the value of the numerical system, as a test of the correctness or erroneousness of our opinions, observations too accordant with the general tenor of our own, to make it requisite for us to quote them.

Mr. Lever has selected as the subject matter of this paper, cases of undoubted "Carcinoma," a disease which never fails to develop its malignant and fatal termination. It occurs, he tells us, more frequently than at first thought we might be led to expect; the registers of the obstetric out-patients of Guy's Hospital show that the proportion of cases of carcinoma uteri to cases of other uterine diseases, is nearly as 1 in 7, or 13·5 per cent.

*Age.*—The period of life most obnoxious to the disease is from the 40th to the 50th year. For, there were:—

| | | |
|---|---|---|
| Between | 25 and 30 years | 3·3 per cent. |
| | 30 — 35 | 15· |
| | 35 — 40 | 10·83 |
| | 40 — 45 | 20· |
| | 45 — 50 | 20· |
| | 50 — 55 | 13·3 |
| | 55 — 60 | 14·16 |
| | 60 — 65 | 0·83 |
| | 65 — 70 | 1·6 |
| | 70 — 75 | 0·83. |

*Condition.*—Single women bear a proportion of 5·83 per cent., married women 86·6 per cent., and widows 7·5 per cent., affording a complete refutation of the statement, that celibacy favours the development of the disease.

But are there not many more married than unmarried women between the ages of 40 and 50? If there are, that would materially affect the calculation.

*Ages at Marriage:—*

| | | | |
|---|---|---|---|
| 34·16 | per cent. married between the ages of | 15 and | 20 |
| 36·66 | .. .. .. .. .. .. .. | 20 | 25 |
| 20·83 | .. .. .. .. .. .. .. | 25 | 30 |
| 1·66 | .. .. .. .. .. .. .. | 30 | 35 |
| 0·00 | .. .. .. .. .. .. .. | 35 | 40 |
| 0·83 | .. .. .. .. .. .. .. | 40 | 45 |

*Number of Children.*—The number of children produced by 113 married women amounted to 596, of whom 301, or 50·51 per cent., were boys, and 295, or 49·49 per cent., were girls. If from these 113 married women, we deduct 10 who had borne no children, we have 103 bearing women, to produce 596 children, rather more than 5¾ to a marriage. One of the sterile women married under 20 years of age, seven under 30 years; one under 40 years, and one at the age of 44 years. Ten barren women out of 113 will amount to 8·8 per cent. This per centage exceeds the average of barren women, for it has been found that 1·20th, or 5 per cent., of married women, are wholly unprolific.

*Abortions.*—Forty per cent. of the females had miscarried. The collective total of their abortions were 122, an average of rather more than 2·54 to each woman. Adding the miscarriages to the number of children born, we shall have the number of conceptions, and we shall find, that out of 100 conceptions, the abortions were nearly 17, or, more correctly, 16·8.

*Complexion.*—The number of light complexioned women affected with this malady, amounted to 20·8 per cent., while those who are recorded as being of dark complexion, amounted to 79.16 per cent.

*State of Uterine Health in early Life.*—Only 25 women, or 20.8 per cent., had enjoyed good uterine health in early life, while 95, or 79·16 per cent., had suffered either from (what is termed) functional disease, or syphilis. The most frequent malady was dysmenorrhœa, with which no less than 66 had been afflicted.

The proportions stand thus:—

| | per cent. |
|---|---|
| Those who had had good uterine health .. .......... | 20·8 |
| Those who had suffered from amenorrhœa .......... | 15·8 |
| Vicarious menstruation .. | 0.83 |
| Menorrhagia ........... | 1·66 |
| Dysmenorrhœa.......... | 54·16 |
| Syphilis .............. | 6.6 |

*Duration of the Disease, and Termination.*—Death occurred in 107 cases, the average duration of the diseases being 20¼ months. The shortest duration of the disease was 3 months, the longest 66 months. In single women the average duration of life was 21·66 months; while in married women, who had borne no children, it was 21·3 months. In females who in earlier or later life had suffered from dysmenorrhœa, the average duration was 19·7 months; in those who had suffered from syphilis, 21 months; from amenorrhœa, 22·9 months, while in those who in early life had enjoyed good uterine health, it was 22·59 months.

Mr. Lever adds:—

"In six patients only have I been able to trace the existence of carcinoma in the maternal parent, four were affected with carcinoma uteri, and two with carcinoma mammæ; this, however, must not be regarded as an average or proportional number, for many women could give no account of their parents, while others stated their mothers to have died from diseased womb, without being able to state the precise disease." 272.

A valuable paper, which induces us to hope that Mr. Lever will continue to pursue, and not forget to promulgate, his researches.

IX. Cases of Measles, occurring oftener than once in the same Individuals. By John Webster, M.D., Consulting Physician to the St. George's and St. James's Dispensary.

Though, in the great majority of cases, measles attack the same individual only once in his life, yet several exceptions to this law have been recorded, and Dr. Webster communicates two which occurred under his own observation, with one which was seen by Dr. Forbes.

*Case* 1.—Master F. H., in the early part of 1837, being then two years old, had a regular attack of measles, along with his elder brother and a cousin, all then living in the same house. I attended him throughout the disease, which exhibited the usual and unequivocal symptoms of that complaint: the eruption was well marked, copious, and continued apparent on the skin for the ordinary period; whilst the affection of the eyes, of the air passages, the cough, and the violence, as well as the duration of the febrile symptoms, placed the nature of his malady beyond any doubt; from which however, in due time, my patient recovered completely, by ordinary antiphlogistic treatment.

On the 23rd of March last, that is, about two years after the first attack of measles, Master F. H. became restless and slightly feverish; but it was only two days afterwards, or the 25th, when an eruption having appeared on his neck and face, the exact character of this patient's complaint remained no longer doubtful. He now had considerable fever, with quickness of pulse, along with the commonly remarked catarrhal symptoms, such as discharge from the nose, watery eyes, and slight cough; he complained likewise of thirst, and he had a foul tongue. On the 26th the eruption continued copious on the arms, legs, and body, at the same time appearing in clusters, slightly elevated, and quite characteristic of its nature; the face, also, was now a little swelled, whilst the catarrhal affection remained; and had any uncertainty yet existed, regarding the nature of this eruptive disease, it would have been entirely removed from the mind of the most sceptical observer by examining a case exhibiting, as this one did, so many well-marked symptoms. The eruption, on the 27th, became less evident; and, by the evening of the 28th, it had almost disappeared, the patient being otherwise much better. On the 29th, Master F. H. was nearly convalescent; and, by the 31st of March, he got quite well." 248.

Of the next instance we may content ourselves with remarking, that the patient, a young lady, passed through the measles at Madras, in 1826, when four years old—a second time, at Blois, in 1832—and a third time, under Dr. Webster's personal inspection, in London, in 1837.

Dr. Forbes attended Lord ———, then ten years old, for measles in 1835, and in 1839, he attended him for measles again.

### X. REMARKABLE CASE OF DRY GANGRENE, OCCURRING IN A CHILD THREE YEARS AND SEVEN MONTHS OLD. By SAMUEL SOLLY, F.R.S. &c.

*Case.*—On the 29th January, 1839, Mr. Solly visited, with Mr. Bury, of Farnham, William Chandler, aged three years and seven months. He had been weakly from birth. His father is a bargeman, earning about a pound a-week. His mother was a healthy-looking woman, but delicate; she has had four children, the eldest seven years old, perfectly well; the second died at two months; the third, William Chandler, is the subject of the case; the youngest was weakly, and since dead. The general food of the family has been bread and potatoes, with meat two or three times a-week.

Mr. Solly thus describes his state:—

"His right fore-arm was gone, having been partially amputated by nature at the elbow-joint by the disarticulation of the radius, though the ulna had been divided lower down, opposite a point which might be seen in the detached radius, as at that place the bone was thinner, a portion of its surface having been removed by absorption, where the line of separation had first commenced. The slough had extended above the elbow-joint, where nature appeared to be making a second attempt at amputation, from the distinct though comparatively pale line of ulceration between the living and dead portions of the limb.

The whole of the left fore-arm, and about half the upper arm, was in a state of dry gangrene; but there was a distinct line of separation in the upper arm.

The foot of the left leg was completely removed just above the ankle-joint, between the epiphysis and the shaft of the tibia and fibula, leaving the extremities of the bone exposed, the soft parts presenting a surface healthy and granulating in one part, but sloughy in another.

On the right foot the phalanges of the second and third toes had been removed by the same unsparing hand, the stumps having a healthy cicatrix; but on the calf and knee of the leg there were livid spots. Such was the extraordinary appearance of this poor little child the first time I saw it. The pulse in the carotids was 140, feeble, easily arrested, but distinct to the eye.

The action of the heart more feeble than natural, but unaccompanied by any unnatural or irregular sound. The mother observed that, when every thing was quiet, at night, it sounded as loud as the ticking of a watch.

The intellects were perfect, and the child, on the whole, wonderfully quiet and tranquil, but complains much of feeling cold unless close to the fire. From the mother's account I learned that the disease commenced at the latter end of last August, both his feet becoming of a blackish purple colour; that hot flannels relieved them only for a short time; sloughs, called by the woman *set-fasts*, began about the beginning of September, on the right leg first, above the heel. These sloughs separated. The sore healed in about a month; that on the left leg never healing, but gradually extending over the foot, and a line of demarcation was ultimately set up. Amputation gradually took place, and the limb was entirely removed by December the 30th.

The disease commenced in the left upper arm, about the middle of November, on the dorsal surface of the wrist. It followed on the right about a fortnight afterwards, commencing below the elbow, and the ulna was divided on the 18th of January; but nature, apparently not being able to effect the amputation perfectly in that spot, recommenced above it. And the radius, together with the hand, was separated at the elbow joint on the 26th January. I could not ascertain the date of the amputation of the two toes on the right foot." 258.

The left arm separated on the first of February, about midway between the shoulder and elbow. The soft parts of the right arm came away on the 5th instant. The dead parts attached to the left leg were thrown off on the 18th. The reparative process proceeded well, and Mr. Solly writes thus:—

"On the 22nd of April I again visited the case. When I found, to my astonishment, that nature, unassisted by the surgeon, had amputated the three extremities, and formed stumps, which might shame many formed by the operator's knife. The stump of the right arm was as perfect as could be desired; the bone in the left projected slightly, but was in the course of removal by absorption. The stump of the left leg was nearly healed, with a very slight granulating projection opposite the bone. The child's health was much improved, his spirits good, and he was freer from pain.

In this case the progress of the disease seems to have been identical with that which is usually observed in senile gangrene; for instance, its first appearance was a dark coloured spot, unaccompanied with pain or swelling, or increase of heat in the part; and very slight redness at the line of demarcation, between the healthy and diseased portions of the limb.

Dr. Carswell, in his admirable article 'Mortification,' in the Cyclop. of Medicine, observes, 'that it is stated, by pathologists, that this form of mortification sometimes occurs in young persons;' but that he himself has never seen it in young persons, and considers it a statement which has not been supported by facts, and I have not myself been able to meet with any case which bears any analogy to it." 266.

A striking case certainly.

### XI. Case of Congenital Absence of the Pericardium, with Observations. By T. B. Curling, Assistant Surgeon to the London Hospital.

*Case.*—The subject of this uncommon malformation was a gardener, aged forty-six, who was admitted into the London Hospital under the care of Dr. Gordon, January 28th, 1839. After exposure to wet he had been seized with symptoms of paralysis, which commenced in the lower extremities, and gradually extended upwards until, the muscles of respiration becoming affected, asphyxia ensued, and he died on the 18th of February. He had previously enjoyed a good state of health, but, before the age of puberty, had been subject to attacks of profuse epistaxis. Nothing remarkable was observed in the action of the heart, or in the pulse.

"The main morbid appearances consisted of ramollissement of the central part of the whole medulla spinalis, deep injection of the mucous membrane of the bladder, and prostatic and membranous parts of the urethra, suppuration of Cowper's gland, and deposits of lymph in the substance of the testicles, with adhesions of the tunica vaginalis. On opening the cavity of the chest, by raising the sternum with the costal cartilages, I was much astonished at finding the heart completely exposed, lying loose in the cavity of the left pleura, in immediate contact with the lung, without any appearance whatever of pericardium. The heart was rather large and flabby, and in its natural position in the chest. On the anterior part of the left ventricle there was a small white opaque patch, and there was another of larger size on the posterior surface of the right ventricle. Nearly the whole front of the right ventricle was coated with a layer of fat. The vessels connected with the heart exhibited nothing remarkable, their relation being perfectly natural. On introducing the little finger into the aorta, its valves appeared to be healthy. The heart was a little overlapped by the anterior edge of the inferior lobe of the left lung, to which it was connected by a slight adhesion about a quarter of an inch in length. The pleura covering that part of the left lung in contact with the heart, was opaque, white, and thickened. The pleura was easily traced from the left lung, along the large pulmonary vessels, to the heart, which it invested so as to form a reflected pericardium, thence passing over the pulmonary vessels on the right side, and the commencement of the aorta, it went forwards to join the pleura costalis, being at first in close relation to the right pleura, to which it was connected by loose cellular tissue, but afterwards separating to leave space for the anterior mediastinum. On the right side of the heart, just above the junction of the inferior vena cava, and close to the diaphragm, there was a pouch in the serous membrane, with a defined margin inferiorly, into which the appendix of the auricle projected. The left phrenic nerve crossed the trachea opposite the first bone of the sternum, and, passing to the front, and rather to the right side of the heart, proceeded to the diaphragm along the anterior mediastinum. The right phrenic nerve passed between the two layers of the separate pleuræ, about two inches behind the left. The lungs were healthy, with the exception of a little emphysema at the margins of the lower lobes. There was an old adhesion half an inch wide, connecting the outer part of the upper lobe of the left lung to the pleura costalis."

This account is very explicit and satisfactory.

### XII. CONTRIBUTIONS TO THE PATHOLOGY OF THE SPINAL CORD. By WILLIAM BUDD, M.D.

The object, a laudable one, of Dr. Budd, is to illustrate the operation of the " reflex function" of the spinal marrow in man, in the same way as it is exhibited in decapitated animals. This he does by a series of cases.

*Case* 1.—John Lang, aged 43, and of temperate habits, without known cause gradually became crooked in the back, and paraplegic, at the age of 18; but regained perfect use of his limbs at the end of two years. The paralysis returned twice; first after an interval of twelve years, and again after two more: each time it followed protracted exertion, but did not continue longer than a few weeks. It returned for the fourth time, and had continued fifteen months, when Dr. Budd first saw him. The curvature of the spine had not increased since the first illness, and in the intervals mentioned the health was quite good. There never had been, nor was there subsequently, any indication of abscess. In the first three attacks sensation was nearly extinct in the palsied limbs, but they had never exhibited involuntary movements. Dr. B. first saw him in July, 1836; and between that time and April, 1837, visited him many times, and took the following notes of his case:—

"Sensation in the lower half of the body begins to fail at the umbilicus, and is very much impaired immediately below it: in the lower extremities it is nearly extinguished; when these are smartly pinched, the patient perceives the nature of the act, but the sensation is very feeble. Has no power of voluntary motion over his lower extremities; once or twice, while he was making strong efforts of the will, I observed a very limited motion of the great and second toes; but had I not watched very narrowly, these movements would have escaped my notice: after having occurred once, or at most twice, he could not repeat them.

These are the only voluntary movements of the lower extremities which the patient can execute. When, however, any part of the skin is pinched or pricked, the limb that is thus acted upon jumps with great vivacity: the toes are retracted towards the instep, the foot is raised on the heel, and the knee flexed so as to raise it off the bed: the limb is maintained in this state of tension for several seconds after withdrawal of the stimulus, and then becomes suddenly relaxed. Once or twice, while the left leg was thus excited, the great and second toes of the right foot were simultaneously drawn towards the sole: the converse never occurred, and, in general, while one leg was convulsed, its fellow remained quiet, unless stimulus was applied to both at once. The contractions excited by stimulating the left leg are uniformly more vigorous than those excited in the right by a like stimulus. In these instances, the pricking or pinching was perceived by the patient; but much more violent contractions are excited by a stimulus of whose application he is perfectly unconscious.

When a feather is passed lightly over the skin in the hollow of the instep, as if to tickle, convulsions occur in the corresponding limb much more vigorous than those induced by pinching or pricking; they succeed one another in a rapid series of jerks, which are repeated as long as the stimulus is maintained. In these experiments the patient is quite unconscious of any thing touching his limb. When asked if he feels anything, his invariable reply is, 'No, I feel nothing, but you see my leg feels well enough.' When any other part of the limb is irritated in the same way, the convulsions which ensue are very feeble, and much less powerful than those induced by pinching or pricking. No movements are excited in his lower extremities by tickling any point in the upper half of his body, though such tickling occasion laughter.

Convulsions, identical with those already described, are at all times excited by the acts of defecation and micturition. At these times the convulsions are much more vigorous than under any other circumstances, insomuch that the patient has been obliged to resort to mechanical means to secure his person while engaged in these acts. To this end two large loops of saddlers' webbing are nailed to the floor; into these the feet are inserted; while two longer loops, also nailed to the floor, are adjusted over the knees as soon as the patient is placed on the night-stool: an attendant is also required to hold down the thighs. During the act of expulsion the convulsions succeed one another rapidly, the urine is discharged in interrupted jets, and the passage of the fæces suffers a like interruption. The convulsions become more feeble towards the completion of the act, and the stream of urine becomes continuous. In the case of making water, the convulsions are uniformly more vigorous the more the bladder is distended. When sufficient precautions had not been taken to secure his person, it had occurred to the patient to be thrown violently forwards on the floor. The passage of urine is felt by the patient, but the sensation is of a smarting kind. Slight involuntary contractions occur in the legs whenever the bladder is distended, and also when the desire to relieve the rectum is manifested. In all these circumstances, the convulsions are perfectly involuntary, and he is unable, by any effort of the will, to control or moderate them. The passions have no power of exciting convulsions. After the lower extremities have been much excited a sense of tingling in them is experienced. Motility and sensation in the upper half are unimpaired, and quite natural. Appetite and digestion pretty good; loss of flesh inconsiderable, and not progressive; nutrition of the legs good." 158.

In the Spring of 1837, Lang regained voluntary power in his lower extremities, with some increase of sensation. As voluntary power augmented, the susceptibility to involuntary movements, and the extent and power of these diminished.

Dr. Budd observes :—The interest of this case lies in the coincidence of these two circumstances : total loss of voluntary power in the lower extremities,—involuntary muscular contractions, excited by application of stimulus to the skin or mucous membranes. There can be no doubt that these contractions were of the same nature, and excited by the same process as those observed in a decapitated animal. Although sensation was never quite extinct in Lang's lower extremities, it was evidently not concerned in the production of the involuntary contractions ; for when an artificial mode of stimulus was employed, the convulsions were uniformly the most vigorous in that case in which the application of it was not felt by the patient, namely, when the hollow of the foot was lightly touched with a feather. Here then we have oral evidence from the subject of experiment, that these acts are quite independent of sensation and volition. The fact that such vigorous convulsions were excited by touching the hollow of the foot with a feather, is of great interest, and tends to show that the nerves which transmit these impressions to the spinal marrow are,—if not the same with those of true sensation,—most actively excited by a stimulus which produces strong impressions on the sensitive nerves. Every one knows how urgent is the sensation of tickling in the hollow of the foot.

In a case, somewhat similar, (Dr. Budd's *second*) the convulsions caused pain.

*Case* 3.—A sailor, aged 17, who had been struck on the back by a boom, presented these symptoms :—

Sensation in the lower extremities unimpaired ; voluntary motion abolished in these limbs, which are rigid, from permanent contraction of the muscles. The legs are sometimes extended, sometimes bent upon the thigh, and the transition from one condition to the other is quite involuntary. When these limbs are in extension it requires great force to bend them in the slightest degree ; and when that force is withheld, they immediately return to their former position ; the converse holds when the legs are flexed. When any part of the skin of the lower extremities is pinched, the corresponding limb jumps with great vivacity; if previously in extension, it momentarily assumes a state of flexion, and *vice versâ*. Occasionally, when one leg is thus excited, its fellow is, at the same time, similarly affected, but not so powerfully. The muscular contraction thus excited is very powerful; it is quite involuntary, and cannot be modified in any way by influence of the will. When any other part of the body is pinched no analogous movements are induced ; but when the patient voids his urine, both legs are always affected in the same way as by pinching their integument. When the legs have been much stimulated by pinching, they become affected with a quivering motion, which lasts some minutes : this also occurs after micturition ; no pain whatever in the legs, nor sense of fatigue ; calves stout and developed; dejections voluntary. There is curvature of the spine, formed by prominence of the dorsal vertebræ, from the fourth to the ninth, inclusive. Motility and sensation perfectly natural in all parts of the body, other than the lower extremities. General health not much impaired. Soon after this the legs became less rigid, and on the 24th of March were observed to be quite placid; convulsive movements could still be excited, by pinching or tickling, but were much more feeble than before.

He died of phthisis, about eight months after receiving the injury.

*Dissection.*—Prominence of the dorsal vertebræ from the 5th to the 9th, inclusive—laminæ somewhat softened, especially on the right side—a little pus external to the dura mater—dura mater there dark and vascular, distended with serous fluid below—diameter of the cord small there—vessels of the pia mater congested above and below the curvature.

The cord itself was of natural size; and a portion, about two inches in length, corresponding to the curvature, softened in the posterior columns. The tissue was not difluent, but became flaky and partially dissolved, when a small and gentle current of water was poured on it. This did not happen when a like current was similarly directed on other portions of the cord. This breaking down of the tissue was much more marked in the posterior than in the anterior columns, which were scarcely, if at all, softened, and resisted considerable traction. After the arachnoid had been removed, the posterior columns had still sufficient consistence to retain their form.

The affected portion was quite white, exhibiting no vascularity or bloody points. The nerves within the vertebral sheath, arising from the softened part, were firm, and of natural appearance. The cord, above and below, perfectly healthy, firm, and of good colour. When the contents of the chest were removed, an abscess was found, surrounding the bodies of the diseased vertebræ, its anterior wall being formed by the aponeurosis of the vertebræ.

We think it cannot fail to strike the reader that the *posterior* columns of the medulla were diseased, while sensation was *unimpaired.* How this quadrates with our notions of the functions of those columns, we do not clearly perceive.

Extensive reflex actions, Dr. Budd goes on to state, are the most striking symptoms of apoplexy of the pons varolii. M. Cruveilhier quotes a case from Ollivier.

> "A man, at work in the fields, complains all at once of acute pain and buzzing in his ears. He immediately runs off, as if to escape from threatened danger. He drops, and there is loss of consciousness; frequent, irregular, and noisy breathing; sometimes stertorous. He sneezes twice with violence; there is stiffness and convulsions of the limbs, alternating with a state of collapse; froth at the mouth, and an epileptiform condition. He dies at the end of five hours, and was not observed the last two. It was not determined whether or not sensation was abolished, but a convulsive movement of the right arm was observed when the skin of that limb was pinched, and a similar movement at the moment when the integuments were pierced in venesection." 167.

On examination after death, the pons varolii was found converted into a sac, full of blood, in part coagulated. The blood had found its way to the surface of the pons by a small lateral opening, and also into the fourth ventricle, which was distended with it.

M. Ollivier states that he has since seen many cases of apoplexy of the pons, at the moment of attack, and has always remarked convulsions of the extremities.

Dr. Budd relates an interesting but a long case, too long indeed for our pages, and too closely connected in its details to admit of useful condensation. But we must observe that, to our mind, it goes far to prove the imperfect condition of the theory of "the reflex function," a theory which we fear is yet to be established in many particulars, and is scarcely adequate to the explanation of all that it professes to embrace.

Dr. Budd reports another case, highly interesting also. But we must pass by its details and content ourselves with the summary he furnishes of the principal facts contained in all the cases, and of the inferences that may be drawn from them.

1st. That the involuntary movements excited in the palsied limbs, were the same in kind as those observed in decapitated animals.

2nd. That these movements were quite independent of sensation, and that they varied in extent and force, inversely with the degree of voluntary power possessed by the affected limbs; being the most forcible when the loss of voluntary power was complete, and diminishing gradually in extent and force as that power increased.

3rd. Irritating one leg generally caused movements in that leg only; in some cases it caused movements, but more feeble ones, in the other leg also, and in the arms when they too were paralysed: facts completely analogous to those observed in decapitated animals.

4th. Impressions on the soles were more efficient than any that were tried on other parts of the skin in exciting involuntary movements; and heat, although not producing sensation, was a powerful means of excitement.

5th. In one case, impressions on nerves arising above the seat of injury, excited convulsions in parts receiving their nerves from below; a fact which proves, that influences from impressions received on incident nerves, at any point, are diffused through the cord. This leads Dr. B to remark that convulsions are rarely, if ever, spontaneous, in the strict sense of that word, for the multitude of impressions constantly received on the surface, especially in the mucous membranes, are probably sufficient, when the motor function of the cord is much exalted, to excite all those convulsions usually called spontaneous.

6th. That the convulsions observed were not associated with a sense of fatigue, but that the power which caused them was subject to exhaustion, and renewed by rest. In cases of injury to the spine, some time elapsed before the palsied parts exhibited involuntary movements; this was probably owing, in some measure, to concussion of the cord, for in decapitated animals these movements may be instantly excited.

7th. Strychnia given internally increased the susceptibility to involuntary movements, and caused spontaneous convulsions that were limited to the palsied parts.

Prussic acid, as well as tincture of digitalis had a decided effect, in one case, of suspending or quieting spontaneous convulsions.

8th. That the dependence of priapism on distention of the bladder, observed in one case, tends to show that external impressions may be reflected by the spinal cord, so as to excite organic processes,—such as, vascular turgescence,—in the same manner as to excite muscular contractions.

Dr. Budd makes some curious concluding remarks:—

"I shall now notice a circumstance in the pathology of tetanus, which gives that disease a link of connection with the preceding cases, and which has been overlooked by many physicians, and has received from none the attention it deserves: I allude to the fact that the convulsions of tetanus, when already present are rendered much more powerful by impressions made on the skin or senses, and when absent, as in the intervals which usually occur, may invariably be excited by such impressions. The fact is stated by Dupuytren in his 'Leçons sur les plaies par les armes à feu,' in the following words:—

'L'intelligence des malades reste ordinairement saine; rarement il survient du délire. Cependant la sensibilité est très developpée, et à mesure que la maladie marche, ou la voit s'accroitre de plus en plus, et se monter à tel point que le moindre bruit, la moindre sécousse, les plus faibles émotions suffisent pour la mettre en jeu et faire entrer le système nerveux, et le système musculaire qui est sous sa dépendance dans un état de convulsion.'

It is therefore of great importance in the treatment of this disease, to avoid multiplying impressions, especially those of a painful kind. In this point of view, the practice of applying leeches repeatedly, and in great number, is very objectionable, for it is not questionable that the evil here alluded to is not compensated by any ascertained advantage. Repeated cupping, blistering, and also repeated purging—means often employed—are open to the same objection. While on this topic, I may be allowed, also, to question the propriety of giving opium in the treatment of tetanus. It has been ascertained that the effect of that drug is to excite, and not quiet, the motor function of the spinal cord; indeed, it is well known that the motor acts of the cord may be rendered much more active and powerful by giving, before decapitation, opium to animals that are to be subjects of experiment. Believing that, after ample trial, experience has not established any advantage in the use of opium, I consider these objections, furnished by theory to be motives sufficient for its future exclusion from the treatment of tetanus. If in the choice of remedies we are to be guided at all by physiological considerations, it is much more rational to seek for remedies for tetanus in that class of substances known to exercise sedative influence on the motor function of the cord. I may mention hydrocyanic acid, conium, and digitalis, as deserving more ample trial than has yet been given them.

Hitherto, the treatment of tetanus has been conducted in violation of all known physiological principles: it is to be hoped that our improved knowledge of the functions of the spinal cord will at length lead us to the discovery of more successful methods." 190.

We are not sure that Dr. Budd's condemnation of opium and of purgatives is quite fair, or, in point of fact, quite correct. If there are two plans of treatment which have proved more successful in chronic tetanus, that is, in curable tetanus, than others, they are—purgatives, and calomel and opium. Nay, we would almost venture to affirm, that opium has been as successful in acute tetanus as any other drug, which, though certainly it does not say much, yet says something in its favour. The hydrocyanic acid, which acts on physiological principles, has been anything but untried, though, as far as we know, it has not proved more serviceable than its rivals.

Dr. Budd reprobates the application of leeches. Certainly local and general depletion have both failed, probably because the hypothesis of inflammatory action on which they were employed, is a deceptive one. But were the convulsions of tetanus attended *with* vascular action, that is, were there any indication for leeches, we should hesitate to discard them in obedience to the excito-motory doctrine, and because they might prove excitants of the convulsions. We suppose that in the convulsions dependent on cerebral congestion in children, leeches would, theoretically, be open to a similar objection; practically, they are useful. It is probable that the general principles which regulate our treatment should, in the present state of the excito-motory hypothesis, be paramount in the application of remedies.

### XIII. MEMOIRS ON SOME PRINCIPLES OF PATHOLOGY IN THE NERVOUS SYSTEM. By MARSHALL HALL, M.D. F.R.S. &c.

Anything which falls from Dr. Hall on the subject of the nervous system deserves and will receive attention. The memoir before us is on the—

*Condition of the Muscular Irritability in Paralytic Limbs.*

Dr. Hall observes:—

"The utmost discrepancy of opinion prevails amongst physiologists and medical writers upon this subject. Prochaska, Nysten, and Legallois state, that the irritability of the muscular fibre remains in paralytic limbs; whilst Professor Müller and Dr. Sticker assert the contrary. No attempt has been made to reconcile a contradiction not very honourable to our science. To explain this discrepancy of opinion is one of the objects of this communication.

The authors to whom I have referred, misled by the generic term and idea of paralsyis, have not sufficiently distinguished between its different species. Yet it will be found, as we proceed, that this distinction is of the utmost importance in the explanation of the phenomena. In fact, cerebral paralysis, or that which removes the influence of the brain, and spinal paralysis, or that which removes the influence of the spinal marrow, are in totally opposite conditions in reference to the irritability of the muscular fibre in the limbs severally affected; facts equally obvious in experiments and in clinical observations." 192.

After quoting the experiments and the opinions of Nysten, Prochaska, Fouquier, and Müller, Dr. Hall dwells on the fact that the convulsive movements occasioned by strychnine are frequently first displayed by the paralytic limbs.

"I figured to myself the fact of the strychnine acting on the spinal marrow, and diffusing its power equally along the nerves, to the right hand and to the left, to the muscles to which they proceed respectively; and I asked myself the question—Is the difference observed in its ultimate effects on those muscles, the power being obviously the same, owing to a difference in the degree of the irritability of the muscular fibre itself? Is the irritability of that fibre actually augmented? If so, the phenomenon would be explained!

I waited with anxiety for opportunities of submitting this question to the decision of experiment. This I entrusted, in the first instance, to my young friend and intelligent pupil Mr. Dolman. The result was as I anticipated. A little child, aged two years, was perfectly paralytic of the left arm. The slightest shock of galvanism was directed to be applied which should produce an obvious effect. It was uniformly observed that the paralytic limb was agitated by a degree of galvanic energy which produced no effect on the healthy limb.

A similar patient, with paralysis of one leg, was subjected to the same experiment by my friend and former pupil Mr. W. F. Barlow, and with the same result.

I repeated the trial on several patients affected with hemiplegia, at my own house, uniformly with the same event: the paralytic limbs were always moved by an influence which was lower than that required to affect the healthy limb, or if both limbs were agitated, it was uniformly the paralytic limb which was more shaken than the other.

I next repeated my observations upon a more extensive scale, at the St. Mary-le-bone and St. Pancras infirmaries. There were two exceptions to the rule; whilst the numbers in which the phenomena already described were observed, were considerable." 201.

Dr. Hall next details some experiments performed by himself. They were made on six frogs. He divided the spinal marrow immediately below the origin of the brachial plexus, and removed a portion of the sciatic nerve of the right posterior extremity. The following were the results.

1st. The anterior extremities alone were moved spontaneously; both posterior extremities remaining entirely motionless, when the animal, placed on its back, made ineffectual efforts to turn on the abdomen.

2nd. Although perfectly paralytic in regard to spontaneous motion, the left posterior extremity, that still in connexion with the spinal marrow, moved very energetically when stimulated by pinching the toes with the forceps.

3rd. The right posterior extremity, or that of which the ischiatic nerve was divided, was entirely paralytic, both in reference to spontaneous and excited motions.

4th. After the lapse of several weeks, whilst the muscular irritability of the left posterior extremity was gradually augmented, that of the right was gradually diminished, phenomena observed when the animal was placed in water, through which a slight galvanic shock was passed accurately in the direction of the mesial plane.

" In this interesting experiment," says Dr. Hall, " we have, then, first the phenomena of loss of spontaneous motion on removing the influence of the brain, the excited or reflex actions remaining; and the loss of these on removing the influence of the spinal marrow; secondly, in the case of mere cerebral paralysis, we have augmented irritability, and in that of the spinal marrow we have the gradual diminution of this property." 202.

5th. Strychnine being now administered, the anterior extremities and the left posterior extremity, or that still in connexion with the spinal marrow, became affected with tetanus; but the right posterior extremity, or that severed from all nervous connexion with the spinal marrow, remained perfectly flaccid.

6th. Lastly, the difference in the degree of irritability in the muscular fibre of the two limbs was observed when these were entirely separated from the rest of the animal.

" In a word," adds our talented author, " the muscles of the limb paralysed by its separation from both cerebrum and spinal marrow, had lost their irritability; whilst those of the limb separated from its connection with the cerebrum only, but left in its connection with the spinal marrow, not only retained their irritability, but probably possessed it in an augmented degree. The next question came to be,—Do these phenomena obtain in the human frame? I visited a patient affected with hemiplegia, including paralysis of the face, and I passed a slight galvanic shock through two pieces of metal, of which one was placed over each cheek. The muscles of the paralytic side were most affected. I repeated the experiment with the same result. I now compared with these, two cases of injury of the facial nerve, passing the galvanic shock in the same manner, through the fibres of the orbicularis: it was now the muscle of the healthy side which was affected by the galvanism, the eyelid of that side being closed, whilst that of the paralytic side gaped as before. I next compared the effect of galvanism in two cases of complete paralysis of the arm, one hemiplegic, the other the result of dislocation of the shoulder. The muscles of the former were more, those of the latter less, irritable than those of the healthy arm respectively, as were also those of the arm of a patient affected with the paralysis induced by lead. Lastly, I compared the cases of paralysis of the lower extremities, one arising after pertussis, and therefore cerebral, the other, I think, from disease within the lumbar vertebræ: in the former there was augmented, in the latter, diminished, irritability.

By means of these experiments and observations we are enabled, I believe, to explain all the apparent discrepancies between the statements of former authors, and between each of them and my own.

The observations of Nysten, and others, determined that the irritability of the muscular fibre still existed in ordinary hemiplegia; but they did not extend far enough to determine the comparative degree of irritability of the paralytic and of the healthy limbs, or the question whether, in the former, the irritability was diminished—the event probably expected—or augmented, a result, I believe, never anticipated.

Prochaska and Nysten and Legallois failed in their experiments, too, by not allowing time for the change in the condition of the irritability of the muscular fibre to take place.

Professor Müller and Dr. Sticker, on the other hand, did not distinguish between paralysis arising from separation from the cerebrum merely, and paralysis arising from separation from the spinal marrow, a distinction of the utmost importance in every point of view, and that which explains the phenomenon under discussion. The term paralysis has been used by all the authors whom I have quoted, in too general a sense. This is so true that I may affirm that in one kind of paralysis, that which removes the influence of the cerebrum, and which is therefore paralysis of spontaneous or voluntary motion, there is augmented irritability; whereas in the other, that which severs the influence of the spinal marrow, the irritability is diminished or even annihilated.

We may conclude that, in cerebral paralysis, the irritability of the muscular fibre becomes augmented, from want of the application of the stimulus of volition; in paralysis arising from disease of the spinal marrow and its nerves, this irritability is diminished, and at length becomes extinct, from its source being cut off.

We may further deduce, from the facts which have been detailed, that the spinal marrow, and not the cerebrum, is the special source of the power in the nerves of exciting muscular contraction, and of the irritability of the muscular fibre; that the cerebrum is, on the contrary, the exhauster, through its acts of volition, of the muscular irritability.

As a further deduction from the same facts, we may infer the diagnosis between cerebral and spinal paralysis: mere cerebral paralysis is attended by augmented irritability, whereas spinal paralysis is that which is attended by diminished irritability. This fact will prove useful in many obscure cases." 206.

There can be no doubt of the value of these facts if they should be completely established. Dr. Hall observes, that the principle they establish explains a whole series of phenomena. And first, he says, the exception to the rule of augmented muscular irritability in paralytic limbs, is obviously dependent upon its existing in the cases of paralysis from the severed influence of the spinal marrow, as distinguished from those arising from the severed influence of the cerebrum merely.

Secondly, we understand at once why the influence of strychnine is first, and most, seen in cerebral paralysis, in the paralytic limbs.

But Dr. Hall brings some other points before our notice.

The first of these is the influence of emotion in paralytic limbs.

The second is the similar influence of certain acts of respiration; as yawning, sneezing, coughing, &c.

The third, the similar influence of the tonic power.

"It must have occurred to us all to observe the influence of surprise or agitation, on the arm and hand, and perhaps on the leg, of a patient long affected by hemiplegia, whilst the limbs of the healthy side remained unaffected. In this case the influence of the emotion is, like that of strychnine in the case formerly discussed, exerted equally upon the limbs of both sides; but it is the muscles of the paralytic limbs which are most irritable, most susceptible of the stimulus; it is therefore these limbs which are most convulsively affected.

The same phenomenon is not observed in paraplegia, because the influence of the emotion is cut off from the affected limbs." 207.

He gives this case.

*Case.*—Dr. Hall was called to a patient a short period ago, affected at that moment with bronchitis. He was forty-three years of age; and at the age of twenty-four had been seized with hemiplegia. Recovering from the immediate danger of the attack, he remained hemiplegic, scarcely regaining the use of the hand and arm at all, and only partially that of the leg.

Whenever this patient is excited by meeting an acquaintance, or in any similar way, he has a little strabismus, and the hand and arm are contracted and convulsed in the most extraordinary manner: whenever he coughs, the leg is thrown involuntarily upwards. The arm is severed, as it were, from volition, but affected by emotion.

And Dr. Hall quotes another case communicated by Dr. Abercrombie to the late Mr. Shaw.

"'I had some time ago under my care, a man affected with hemiplegia of the left side; the palsy complete, without the least attempt at motion, except under the following circumstances: he was very much affected with yawning, and every time he yawned the paralytic arm was raised up, with a firm steady motion, until it was at right angles with his body (as he lay in bed on his back), the fore-arm a little bent inwards, so that his hand was above his forehead at its greatest elevation. The arm was raised steadily during the inspiration, and when the expiration began seemed to drop down by its own weight, with considerable force. He continued liable to the affection for a considerable time, and it ceased gradually as he began to recover the natural motion of the limb.' —That is, as I conclude, as the state of augmented irritability was removed by the returning acts of volition." 208.

Not less interesting, continues our author, are the effects of the tonic power. In cases of hemiplegia of long duration, the paralytic limbs, but especially the arms and hands, are drawn into a state of chronic, rigid, contraction. This phenomenon is owing to the principle of tone constantly acting upon muscles now possessing augmented irritability, whilst they are never, or rarely, relaxed by acts of volition.

A similar effect is seen in idiots born with atrophied cerebrum: the influence of volition is wanting; that of the spinal marrow, the source, at once, of the tone and of the irritability of the muscular system, is in constant action, and induces chronic contraction, an effect which must, however, be distinguished from that of spasm, which is excited immediately by some disease of the spinal marrow itself.

Dr. Hall introduces eight other cases with the following remarks :—

"I may now resume the subject of the action of strychnine on paralytic limbs. It is obvious that the generalization of M. Fouquier, M. Ségalas, and others, that the strychnine attacks the paralytic rather than the healthy limbs, was too hasty. This is only true in those cases of paralysis in which the muscles still remain in nervous connexion with the spinal marrow; the opposite result is observed in those other cases in which such connection between the muscles and the spinal marrow is intercepted.

I would here make another observation. The arms and hands, generally speaking, are more under the influence of the cerebrum than the lower extremities; and these, on the other hand, are more under the influence of the spinal marrow than the arms and hands. The superior extremities are more and more frequently affected by hemiplegia than the inferior; these are more influenced by tetanus, by strychnine, &c., than the former, a fact which I have observed, in regard to strychnine, in some cases of hemiplegia. These facts must be borne in mind in making our observations.

Another circumstance must also be noticed. The more perfect the paralysis, generally speaking, the more the irritability of the muscular fibre is augmented. In hemiplegia, the arm is generally at once more paralytic and more irritable than the leg. In chronic cases, however, the irritability becomes impaired, together with the nutrition." 210.

And, finally, Dr. Hall concludes :—

"1st. That the spinal marrow, exclusive of the cerebrum, is the source of the muscular irritability :

2nd. That the cerebrum is, in its acts of volition, an exhauster of that irritability :

3rd. That, in muscles separated from their nervous connexion with the brain, we have augmented irritability :

4th. That, in muscles separated from their nervous connexion with the spinal marrow, we have on the contrary, diminished irritability :

5th. That the degree of the irritability of the muscular fibre of paralytic limbs, compared with that of the muscles of the healthy limbs, will afford us a source of diagnosis between cerebral and spinal paralysis, and especially between

{ 1. Hemiplegia of the face, and
{ 2. Paralysis of the facial nerve;

{ 3. Hemiplegia of the arm or leg, and
{ 4. Disease of the nerves of these limbs ;*

{ 5. Disease of the spinal marrow in the dorsal region, and
{ 6. Disease of the cauda equina in the lumbar region ; &c.

6th. That the greater influence of emotion, of certain respiratory acts, of the principle of tone, &c., on the muscles of certain paralytic limbs, than on those of healthy limbs, depends on their augmented irritability :

---

* "In disease of the cervical vertebræ, the arms are sometimes paralyzed without paralysis of the legs; this probably arises from compression of the brachial plexus. See Sir B. Brodie's paper in the transactions of this Society, vol. xx. p. 130; the galvanic trough would determine the question."

7th. That the same principle explains the greater susceptibility of the muscles in certain cases of paralytic limbs, to the influence of strychnine.

8th. That, in the conclusions of M. Fouquier, Professor Müller, &c., a sufficient distinction was not made between the influence of the cerebrum and of the spinal marrow, which in this, as in so many other respects, have such different properties:

9th. From these, and other experiments and observations, I conclude, too, that sleep restores the irritability of the muscular system, by arresting the acts of volition, which exhaust or diminish it; muscular efforts, on the other hand, diminish the irritability and induce fatigue." 217.

Undoubtedly a valuable memoir.

### XIV.—Case of Fracture of the Coracoid Process of the Scapula, with Partial Dislocation of the Humerus forwards, and Fracture of the Acromion Process of the Clavicle. By John F. South, Assistant Surgeon to St. Thomas's Hospital.

Mr. South publishes this case because the occurrence of such an accident has been doubted, and no dissection of it, so far as he knows, has been made.

A painter, aged 58, was admitted into St. Thomas's Hospital, at 7 p. m. April 12, 1838, having fallen from a height of 30 feet.

There was a scalp wound and bleeding from the left ear, and a compound fracture of the olecranon opening into the joint.

"The arm hung closely to the side, and at first I did not observe any injury of the shoulder; but being informed that the surgeon who had sent him to the hospital thought there was dislocation at that joint, I examined it more carefully, and on rotating the arm, felt crepitus, which I considered to arise from the motions of the broken ulnar being transferred to the humerus. However, not being satisfied on this point, I fixed the olecranon, and then looking more closely at the shoulder, observed a depression below and behind the acromion, which led me to suppose some displacement of the head of the humerus or fracture of the neck of the scapula, but still the roundness of the shoulder was not lost. I then put my hand on the shoulder, and endeavoured to grasp the neck of the scapula with my thumb and finger (the former being in the axilla,) and commenced gentle rotation of the arm, when immediately and unexpectedly I felt the head of the humerus move backwards, and the appearance of pit ceased. I then began to arrange the elbow, and whilst doing this the humerus again slipped out, the same appearance of depression behind it and below and behind the acromion was re-produced; and I also observed there was preternatural prominence and roundness of the front of the shoulder. I therefore supposed it to be dislocation under the clavicle, of the same kind, though not to the same extent, as the so-called dislocation under the pectoral muscle, but which is really rather under the inner edge of the deltoid than under the pectoral muscle.

For the fracture at the elbow joint a long well-padded splint was placed on the front of the limb, to which the fore-arm was connected nearly as high as the elbow by another, and the upper arm in a similar manner, but less tightly, from a little above the joint nearly to the axilla. The edges of the wound were brought together with two adhesive straps, and the plaister varnished with sealing-wax, after Mr. Abernethy's plan, so as to allow the application of an evaporating lotion without the straps being loosened.

The dislocated head of the humerus was now again replaced, merely by lifting the neck outwards with the thumb and rotating the arm: its return was this time indicated by a grating noise, which was heard by the bystanders. A large pad of tow was put in the armpit, and confined there by a bandage tied upon the opposite side of the neck, and a second bandage passed round the belly, and including the fore-arm, brought the band near to the trunk, whilst the head of the humerus was kept up by the pad in the axilla." 105.

We may venture, we think, to make one observation. The facility with which the head of the humerus slipped forwards under the inner edge of the deltoid and back again, might direct attention to the coracoid process. The integrity, or otherwise, of *that*, is usually distinguishable, without much difficulty; for, by firm pressure below the clavicle, the process itself may be, in general, felt with ease, and subjected to examination.

In the night of the 16th, cough and vomiting came on; the vomiting continued, and at 4 a.m. of the 17th, he died.

*Examination of the Shoulder.*

"On turning off the integuments, a small quantity of effused blood was found on the front of the shoulder, and to my surprise, a fracture of the clavicle, about a third of its length from the acromial extremity, with, however, but little displacement. The clavicle was then disarticulated from the sternum, and, together with the scapula and part of the humerus with their muscles, removed from the body and carefully examined. The acromion was broken at the usual place, about an inch from its extremity, but not at all displaced, as the periosteum had not been lacerated.

When the deltoid muscle had been turned off from its clavicular origin, the coracoid process of the scapula was found broken about half an inch from its tip, into two unequal pieces, the smaller of which remained connected above with a piece of the triangular ligament still attached to the acromion, and below to the short head of the biceps muscle, which had pulled it down as far as the ligament would allow, about half an inch below and to the outer side of the stump of the coracoid process. This muscle was torn from the coracobrachialis about an inch; and to the top of the conjoined tendon of the latter, and of the lesser pectoral muscle, was attached the larger portion of the broken coracoid process, which had been drawn rather lower than the other portion, and to the inner side, but was still connected by a thin cord of fibrous matter to the coracoid process. The remainder of the triangular ligament was torn to pieces. In the front of the capsule of the joint was a slit about an inch in length, through which the cartilaginous covering of the head of the humerus was seen glistening. The remaining origin of the deltoid was divided, and the muscle turned down and cut off, which exhibited the head of the bone in its proper position.

Being anxious, if possible, to reproduce the same state of the shoulder as when I first saw him, in order to comprehend the extent of the displacement, I moved the humerus about in various directions, when at last, by lifting up the shaft and pressing the head of the humerus forwards, I effected my purpose; the preternatural roundness in front of the acromion being produced by the head of the humerus being partially thrown forwards and over the front edge of the glenoid cavity, so that it became fixed, and behind it the depression below the acromion appearing, in consequence of the sinking of the tendons of the infraspinatus and teres minor muscles into the glenoid cavity, from the altered position of the head of the bone, which, however, did not protrude through the slit in the capsule, although it was there more distinctly visible. No other laceration of the tendons about the joint had occurred, except a very slight tearing of the tendon of the supraspinatus muscle. Nor was there any other fracture of bone." 106.

Mr. South, among other observations, makes the following:—

"As to the partial dislocation of the humerus, it is an accident which I have often heard mentioned, but till the examination of the present case have scarcely deemed possible. Indeed, even in this, during life, I did not suppose it existed; and I still think that it could not have occurred without great laxity of the capsular ligament, and without the fracture of the coracoid process. I believe that the existence of the latter injury was necessary to allow the partial dislocation of the head of the humerus forwards; for had the coracoid process remained unbroken, I cannot imagine how the head of the membrane could have slipped so far before the glenoid cavity, as to allow of its being fixed in the position in which I found it when I first saw the patient, and to which I returned it after death." 107.

"I would wish, however, to state, that I do not consider the present case as offering any proof of the possibility of such accident as partial dislocation of the head of the humerus, unless, as in this instance, there be fracture of the coracoid process. For the disposition of that process is not merely as a protection against injury to the front of the joint, but it precludes the head of the humerus being projected so far before the edge of the glenoid cavity as to put it in a situation from which it cannot and must not be immediately withdrawn by the slightest action of either of the muscles attached to the tubercles. Whilst, on the contrary, if the head of the bone be driven backwards, it must be either so completely thrown from the glenoid cavity, as to produce dislocation on the dorsum, or so trifling, that it is but for a moment slightly displaced, and reverts to its natural place so soon as the impelling force is removed." 109.

The surgical world is under obligations to Mr. South for communicating this case.

### XV. Memoir on Tuphlo-enteritis; or Inflammation and Perforative Ulceration of the Cæcum, and of the Appendix Vermiformis Cæci. By John Burne, M.D. Physician to the Westminster Hospital, &c.

Since Dr. Burne laid before the Society, in May, 1836, an article on the Inflammation and Perforative Ulceration of the Cæcum and of the Appendix Vermiformis Cæci, he has seen more of these cases, and acquired additional information respecting them.

"The peculiarity," says Dr. Burne, "in the organization of the cæcum, which bears upon the present subject, is the absence of a peritoneal tunic at its posterior part, where it is fixed and attached by adipose cellular tissue to the iliac fascia, so that in the event of a perforative ulceration in this direction an abscess would form behind, and without the peritoneum upon the iliac fascia, and direct its course to the lumbar region at the outer edge of the quadratus lumborum muscle, as described in the former memoir.

Now of the whole tract of the large intestine, no part is so exposed to the lodgment of undigested substances as the cæcum, owing to its particular conformation, as well as to its being the portion of the large intestine with which the undigested substance comes first in contact. These circumstances, strengthened by the fact that many of the patients attacked with inflammation of the cæcum remember to have swallowed accidentally, or to have taken as part of their ordinary food, matters likely to pass the stomach in an undigested crude state, admit of the conclusion, that the greater number of the cases of inflammation of the cæcum are to be ascribed to the prolonged irritation of bodies so lodged, and that such inflammations are therefore properly symptomatic; a conclusion borne out, moreover, by the manner of attack, which is characterized by the development of the local preceding that of the general symptoms, and by the absence of the chills and rigors, which usher in idiopathic inflammation. The inflammation is developed slowly in one person, speedily in another, owing to the greater or less susceptibility and inflammatory predisposition of the individual. The inflammation of the cæcum may be idiopathic, and arise from the ordinary exciting causes, cold and vicissitudes of the weather, there can be no doubt; though the instances are rare in comparison with those which may be fairly attributed to the irritation of crude substances, which have reached the cæcum and lodged in its pouch.

The termination of this symptomatic inflammation of the cæcum is usually by resolution; the symptoms yielding at the end of five or six days, and subsiding altogether soon afterwards; except in patients of an inflammatory or gouty diathesis, in whom inflammation once excited will continue in a sub-acuto-chronic form, and require several weeks for its removal, notwithstanding the original exciting cause shall have passed away. The termination by perforative ulceration and abscess is rare, and probably never occurs when the cæcum was sound prior to the attack; the open channel of the colon permitting the passage of the irritating body, as soon as the subsidence of the spasm and the removal of the inflammation allow the muscular tunic of the cæcum to resume its function. No instance has come within my observation in which, upon dissection, perforative ulceration of the cæcum has been found; all the cases of inflammation of the cæcum and of supposed perforation, within my knowledge, having recovered; and, in the cases on record, there is evidence of disease in this gut having existed previously." 36.

Dr. Burne relates seven cases. The first, one of sub-acute tuphlo-enteritis,—the second, of acute tuphlo-enteritis—the third, of enlargement of the appendix; sero-enteritis originating in the peritoneal tunic of the appendix —the fourth, of concretions in the appendix vermiformis cæci, pelvic abscess and peritonitis; the appendix being situated in the pelvis—the fifth, of perforative ulceration of the appendix vermiformis cæci; abscess discharged by the bowels; recovery—the sixth, of perforative ulceration of the appendix vermiformis cæci, from a concretion lodged in its canal; peritonitis; death —the seventh, of perforative ulceration of the appendix vermiformis cæci; circumscribed abscess; death; unusual situation of the appendix.

These cases are all possessed of interest, and tend to exhibit the main features of the affection. But we cannot afford space for their details, and pass to the observations which follow them.

The varieties of disease, says Dr. Burne, in the present and former memoirs consist of:—

1. Inflammation, acute or sub-acute, of the cæcum, terminating quickly or slowly in resolution, or lingering on and leading to permanent organic impairment.

2. Perforative ulceration of the cæcum from within, with abscess behind the peritoneum, pointing externally in the corresponding lumbar, or inguinal region, or in both.

3. Inflammation of the appendix, spreading over the peritoneum.

4. Perforative ulceration of the appendix, with consequent universal peritonitis, ending rapidly in death; or with circumscribed peritonitis and abscess within the peritoneum, sometimes ending in death in the course of ten days, or, life being preserved, bursting eventually into the cæcum, and discharging itself by the rectum, or directing its course to the surface of the body, and pointing in the right lumbar or inguinal region. If the appendix happen to be situated anormally, then the locality of the abscess, always within the peritoneum, is determined accordingly; as also is the direction it takes, or in which it points.

Dr. Burne adds:—

"On referring to the Leçons Orales of Dupuytren, and to the article of Mr. Ferrall, on Phlegmonous Tumours in the right iliac region, I find that neither of these authors have spoken of the appendix. The greater number of cases detailed by Dupuytren as original phlegmonous tumours and abscesses in the right iliac fossa, appear to have been cases of perforative ulceration of the appendix, the tumours and abscesses being consequent on this perforation, and not orignal. The appendix, indeed, seems to have been overlooked in the necrotomy, no mention being made of it. It has also been overlooked by others, under similar circumstances; by Ferrall, Menière, Husson, and Dance, and it is the pathological error which has resulted that gives an especial interest to the subject." 59.

After criticising the opinions of Dupuytren, that inflammation of the right iliac fossa depends on the presence of irritating matters in the cæcum—and the *pendent* opinion of M. Menière, that it results from the extension of inflammatory action from the mucous membrane to the cellular, Dr. Burne, goes on to remark that Dupuytren, Menière, Husson, and Dance appear to him to have confounded together the four varieties of disease above set down, and have mistaken them for a primary inflammation and abscess of the subcæcal tissue; while the abscess itself is secondary, is only a stage or symptom, and results from the perforative ulceration either of the cæcum or of the appendix.

Speaking of the tumor which occurs in the right iliac fossa, Dr. Burne says that he has no difficulty in assigning as its causes:—

1st. Collection of fæcal matter in the cæcum.

2nd. The presence of any crude, undigested substance, of worms, concretions, or other foreign bodies.

3rd. Inflammation of the cæcum, resulting from the irritation of the above.

4th. Chronic disease of the cæcum, as in cases III. and IV. of my former memoir.

5th. Abscess from perforative ulceration either of the cæcum, or of the appendix.

In reference to diagnosis, Dr. Burne concludes that it is not difficult to distinguish the cæcum and appendix cases from other diseases, although it is by no means easy to distinguish the varieties from one another. He offers the following suggestions on this head:—

"The inflammation of the cæcum, tuphlo-enteritis, the most frequent variety, is distinguished by the sudden attack, the patient having been in health previously; by the local signs fixed in the right iliac fossa; by the vomiting and obstinate constipation; and by all these signs being effectually relieved, or passing entirely away as soon as a free action of the bowels can be procured. This variety is the least dangerous.

The perforative ulceration of the cæcum, which is comparatively rare, may be suspected when a patient, who has been in ill health, and labouring under bowel complaints for some time, is seized suddenly with the local signs fixed in the right iliac fossa, together with constipation and vomiting; the local signs continuing to persist in all their urgency, notwithstanding the constipation shall be overcome, and the free action of the bowels re-established. Later it will be distinguished by abscess without the peritoneum, pointing in the right groin, or in the right lumbar region, at the outer edge of the quadratus lumborum muscle; the tumour of the abscess being not unfrequently emphysematous.

Ulceration of the mucous membrane, as indicated by the bowel complaints, is a pre-existing condition of the cæcum, and invading eventually the other tunics, perforates the cæcum at its posterior part, where it is devoid of peritoneal tunic; hence the inflammation and abscess of the sub-cæcal tissue, which give rise to all the phenomena of phlegmonous tumours, or abscesses in the right iliac fossa. Perforative ulceration of the anterior or lateral parts of the cæcum, covered by peritoneum, rarely happens. I am not acquainted with any case of this description, as proved by dissection. It would necessarily excite peritonitis, which, if circumscribed, would lead to abscess; if universal, would speedily destroy life. These characteristics render the distinction between the perforative ulceration, and the simple ulceration of the cæcum very obvious.

The inflammation of the appendix is very rare, one case only having occurred in my experience. It was confined to the serous tunic, and presented the signs of circumscribed idiopathic peritonitis.

The perforative ulceration of the appendix is next in frequency to the inflammation of the cæcum, being certainly much more common than perforation of the cæcum. This variety may be suspected by the more or less sudden development of the local signs, always severe; by their being fixed in the right iliac fossa, and not preceded by bowel complaints, or ill health; by the supervention of vomiting and constipation, the constipation yielding readily to medicine, yet, having yielded, no amendment following; by the great tension in the ilio-inguinal region, there being always a circumscribed peritonitis and abscess within the peritoneum; by the sympathetic tenderness of the whole abdomen; and, subsequently, by the occurrence of a diarrhœa, and a discharge of pus by the rectum, followed by subsidence of the tumour, and amelioration of all the symptoms; or, by the pointing of the abscess in the form of an emphysematous tumour in the lumbar-inguinal, or ilio-inguinal regions.

The peritonitis, excited at the moment of the perforation of the appendix, will not unfrequently spread rapidly and universally over the peritoneum, and destroy life in from twelve to twenty-four hours.

The absolute perforation of the appendix is preceded by ulceration of the mucous membrane of some standing not resulting from disease of the intestinal canal, but produced by the accidental presence and infraction of some small foreign body: hence bowel complaints do not usually precede the perforation of the appendix.

The perforation of the appendix has many symptoms in common with the perforation of the cæcum. The perforation of the cæcum, however, is generally preceded for weeks or months by bowel complaints, indicating ulceration of the mucous membrane; while the perforation of the appendix is not preceded by such bowel complaints; a difference in the state of the canal prior to the occurrence of the perforation which aids materially in establishing the diagnosis." 68.

And Dr. Burne completes his interesting memoir by the following

*Analysis of Twenty-one Cases.*

Mortality: 13 recovered; 8 died.

Character: 19 acute; 2 chronic.

Varieties: 11 were inflammation of the cæcum—all recovered.

2 were chronic disease of the cæcum—both died.

1 was ulcerative perforation of the cæcum from within, with abscess externally—recovered.

1 was inflammation of the appendix, with circumscribed peritonitis—died.
6 were ulcerative perforation of the appendix—five died, one recovered.

Of the five fatal cases of perforative ulceration of the appendix, one died of diffuse peritonitis in about sixty hours; one of peritonitis, and circumscribed abscess in the peritoneum, in nine days; one of circumscribed peritonitis, and abscess in the peritoneum, in twelve days; one of circumscribed abscess in the peritoneum, in four weeks; and one of abscess in the peritoneum, pointing in the right ilio-lumbar region, in eleven days.

The one which recovered was a circumscribed abscess in the peritoneum, bursting into the cæcum.

Age,—Two under ten years of age: seven between ten and twenty; three between twenty and thirty; six between thirty and fifty; three between fifty and seventy.

Sex,—Sixteen were males; five were females.

Occupation,—Six were gentlemen; one was a coachman; one a farrier; five were boys having no particular occupation; three were destitute; five were females having no particular occupation.

Season,—In the autumn and beginning of winter, more frequently.

Dupuytren, from the analysis of sixteen cases, infers that they occur at the end of summer and beginning of winter more frequently; that age has an incontestible influence, eleven out of the sixteen having been under thirty years of age; that men are more liable than women; and that a greater liability applies to trades, as painters, colour-grinders, and turners in copper.

The inferences from this analysis are borne out by the foregoing in so far as relates to sex and season. They differ in some degree as relates to age; and entirely as relates to the liability of particular trades.

In the twenty-one cases the age varied from six to seventy years; the number under thirty being twelve, viz., four-sevenths of the whole; while Dupuytren's rather exceed two-thirds. The greater susceptibility of the alimentary canal in young persons would predispose them to inflammation of the cæcum, from the irritation of crude, undigested substances, and give a preponderance of cases under the middle age. The liability of particular trades, Dr. B. believes to be fallacious. The twenty-one cases having occurred in persons wholly unconnected with trade. Indeed, the causes being accidental, and having no relation to occupation, leave no ground for the supposition that the kind of trade can be instrumental in producing the various affections of the cæcum and appendix.

### XVI. On the Softening of Coagulated Fibrine. By George Gulliver, F.R.S. Assistant Surgeon to the Royal Regiment of Horse Guards.

Mr. Gulliver's object may be best described in his own words:—

"The nature of many of the albuminous morbid products affords an extremely interesting subject for further inquiry, since it is probable that a great step in the advancement of pathology may be dependent on the acquisition of a more precise knowledge of their distinctive characters. Although these matters differ most remarkably in their consequences, and are apparently developed in the living body under conditions essentially distinct,—seeing that they have properties more or less analogous to those of albumen—M. Andral, and most of the pathologists of the French school, have earnestly insisted on this analogy, with the view of informing us, that the substances in question are nothing but a modification of one of the proximate principles of the blood. It is possible, however, that this kind of generalization may be premature, and it seems doubtful whether, by attaching too exclusive an importance to rather vague generic characters, it may not have tended to retard that more particular examination of these substances, still so essential to a satisfactory knowledge of their intimate constitution; for, in the present state of pathological science, it is with their specific differences that we require to become acquinted; and their affinity to albumen, and common points of resemblance, so far from leading us to this desirable end, present the greatest difficulty in the investigation.

Of all these products, from its constituting one of the most frequent elementary forms of disease, pus is the most important; and as various matters have been comprised under this denomination, some investigation of their peculiar properties would appear to be necessary. Hence any contribution towards this object will not perhaps be altogether unacceptable, and I therefore beg to submit to the Society a narrative of some experiments and observations from which it may be concluded that a fluid which has generally been described as purulent is, notwithstanding, of an essentially different nature. I have elsewhere mentioned incidentally that this matter is softened fibrine, and I now adduce the evidence on which that opinion was founded, together with such remarks as may appear necessary by way of illustration." 138.

Mr. Gulliver relates seven experiments on the concoction of fibrine—four cases of softened fibrine in the heart and arteries—and five cases of softened fibrine in the veins. We shall quote :—

1. *Concoction of Fibrine.*

*Experiment* 1.—A clot of fibrine, such as is so often found there after death, was taken from the heart, and carefully secured in a perfectly clean portion of small intestine turned inside out, so that the fibrine was in contact only with the peritoneal surface of the gut. It was then placed in a water-bath, and kept for forty-three hours at a temperature varying from 96 to 104 degrees, when the fibrine was found to have become partially softened, particularly in some places towards the centre. It had rather an offensive, though not at all a putrid odour, and no ammoniacal gas appeared to be emitted from it, judging from the test of muriatic acid.

About a drachm of the softened fibrine was collected for examination. It could not have been distinguished from pus either by its colour and consistency, or by the facility with which it was miscible with water; it was not rendered ropy by caustic volatile alkali, and presented no irridescence when pressed between plates of glass before a candle in the manner pointed out by Dr. Young. Subjected to the microscope, the fluid was found to be loaded with particles extremely variable in size and form; some appeared very dense, others were flaky and larger than pus globules, but the greater number were smaller, extremely irregular in shape, not at all globular, like pus, but consisting of very minutely granular masses, and a few rounded particles about half the diameter of those of the blood were observed. On the addition of acetic acid all the particles soon disappeared, except a few which seemed more compact and required a longer time for solution. Treated with sulphurous acid, they swelled considerably, and became nearly invisible. Some of the softened fibrine was put into a small vial of water, a similar quantity of the clotted fibrine which had not been concocted being in like manner placed in another vial. Both were set aside for four days, the temperature of the air at the time ranging from 60° to 74°, when the mixture with the concocted fibrine was but very faintly putrescent, and that in the other bottle highly so.

*Experiment* vi.—Some of the fibrinous concretion formed, in consequence of inflammation, on the pleura of a dog, was confined in a glass tube, and subjected to a blood-heat for eighty-four hours, when it was found to be throughout softened, and presenting just the colour and consistency of pus, from which however it was easily distinguishable by the characters noticed in the other trials.

2. *Softened Fibrine in the Heart and Aorta.*

*Case* 2.—J. Cowan, 56th regiment, aged 29, died of pulmonary consumption. Some fibrinous clots were found among the fleshy columns of both ventricles, similar concretions of smaller size were attached to the inner membrane of the descending aorta, and some of the smaller branches of the pulmonary artery were filled with coagula. All these clots were more or less softened in the centre, the surfaces of those in the heart and aorta being toughish and cyst-like. The matter resembled pus in colour and consistency, but differed from it in being composed of very irregular particles and a few extremely minute globules, all readily soluble in acetic acid, swelling and becoming indefinite under the action of sulphurous acid, and not irridescent when placed before a candle. In short, it was destitute of pus globules, and presented just the same characters as the softened fibrine described in the first experiment.

3. *Softened Fibrine in the Veins.*

*Case* 8.—Corporal Pat. Long, aged 35, 86th regiment, died of chronic dysentery; the large intestines were ulcerated, but no collections of pus could be detected in any part of the body. The cava, renal, femoral, and saphena veins were completely filled with a clot, which in the former had become softened in the centre, the fluid being of a reddish-brown colour. It was faintly irridescent, and oberved under the microscope to be composed principally of very irregular particles, similar in all respects to those of the softened fibrine, but pervaded also by a considerable number of globules, somewhat smaller than those of the blood, pretty regular in size, and rough on the surface. On the addition of acetic acid or carbonate of ammonia they instantly disappeared, and the irregular particles were slowly dissolved in the acid.

The preceding experiment and cases exhibit the chief facts, more circumstantially set forth by the remainder.

Mr. Gulliver observes, that most of the cases which have fallen under his notice of fibrinous pulp in the veins have not presented marks of inflammatory action in the coats of the vessels, such as roughness of the inner membrane from adherent coagulated lymph, or increased vascularity or suppuration in their cellular sheaths. The softening of clots of fibrine appears, indeed, to be independent of inflammation, although this process and its consequences may be expected to occur sooner or later in the neighbourhood, and we may accordingly find true purulent fluid mingled with the softened fibrine, just as we often observe pus with cancerous and tubercular matter.

It may be observed that the softening of fibrine certainly occurs sometimes without appreciable suppuration in any part of the body. Mr. Gulliver tells us, that he and Dr. Davy have seen such cases, and we may add that we have done so too. The following remarks agree exactly with our own experience, such as it is and so far as it has gone.

"Although the occasional obstruction of the veins by sanguineous coagula, whether softened or not, has become of late years well known from the labours of the continental pathologists, and particularly in this country from several reports of dissections in the magnificent work of Dr. Bright, as well as from the essays of Mr. Arnott, Dr. Geo. Burrows, and Dr. Copeland, my observations lead me to believe that the affection is not only more frequent than is generally supposed, but more concerned in disease, a circumstance to which my attention was first directed by Dr. Davy. Those who have not been in the habit, when examining dead bodies, of accurately attending to this point, would be surprised at the numerous examples of this condition noted in the register of dissections made under his superintendence at Chatham. The clots were in most instances partially whitened and softened in the centre; they were found in the heart, and also adhering to the inner membrane of the aorta; in the veins of the lower limbs, as well as in the cava, portal, and renal veins; in the pulmonary vessels, and in the sinuses of the dura mater. They were chiefly observed in subjects who having long suffered from various chronic maladies, had great prostration of the vital powers, and languor of the circulation during the last days of existence; a state which was also remarkable in the cases related in this paper, and which seems especially favourable to the formation and softening of the clot. If, therefore, a vein becomes obstructed with coagulated blood or fibrine, which cannot easily be absorbed or organized in consequence of the very feeble vitality of the patient, the clot may be expected to soften, and it is accordingly in such cases that the affection may be often found after death, although never suspected during life." 149.

It is impossible to be much in the dead-house of a large hospital, without being struck with the comparative frequency of obstruction in the iliac and femoral veins. And we have found it, as Mr. Gulliver has, in those who die after a lingering illness, or a protracted final struggle, and in whom the circulation for some period before death must have been extremely languid.

Mr. Gulliver continues:—

"On the contrary, when the organic force is active, we know how readily fibrine becomes the seat of new formations, that it will acquire firmness and remain for years in the sac of an aneurism, and that extravasated blood is generally soon absorbed after injuries. In repeating those experiments of M. Gendrin, in which a seton is passed through a clot of blood, either in or out of the vessels, I could only twice succeed, after numerous trials, in producing any thing like pus in the substance of the coagulum. In these instances the animals were greatly reduced from the effect of previous operations, and the matter proved to be nothing more than softened fibrine, such as I have described it. When blood was injected into the subcutaneous cellular tissue, or into the serous cavities, its complete coagulation was retarded for two or three hours, while some of the same blood kept at the animal temperature in a bottle always coagulated in less than eleven minutes. Absorption of the clot took place with great rapidity, and was hardly delayed by passing a seton through it, so that on the second or third day it had nearly disappeared, the threads approaching the skin, where of course pus would be formed independently of the extravasated blood. When two ligatures were so placed on a vein as to include an inch or two of the column of blood, it took about three hours to coagulate perfectly, as Sir Astley Cooper had noticed in his experiments. In two or three days it lost its colouring matter, becoming whitish, tolerably firm, and shrunken. After a seton or a leaden shot was put into the clot, no appearance of a purulent-like fluid was seen towards the centre, although pus was frequently present between the coagulum and lining membrane of the vessel, and still more often in the cellular coat and the neighbouring parts.

It may be observed, therefore, that as the doctrines of M. Gendrin concerning suppuration, have been extensively promulgated in almost every work on the subject recently published in this country, the preceding cases are interesting in this respect, since they are of that species to which he attaches so much importance, as proofs of the conversion of clots of fibrine or blood into pus. However, as Mr. Palmer remarks, M. Gendrin appears to have confounded the fibrine or corpuscles in a manner not consistent with the known constitution of the blood; and although some purulent or other matters may be occasionally mixed with the softened fibrine, still as it results from several observations that the latter is essentially distinct from pus, and constitutes the whole or greater part of the so-called purulent fluid in these cases, it is at least doubtful whether they admit of the inference which M. Gendrin has drawn from them." 151.

Mr. Gulliver adds these conclusions:—

1st. That coagulated fibrine, when removed from the body and subjected to a blood-heat, commences to soften in about forty hours, assuming the colour and consistency of pus, but easily distinguishable from it by microscopic and chemical examination.

2nd. That the purulent-like fluid found in the fibrinous clots of the heart and arteries, and so frequently in the viens, is essentially distinct from pus, and analogous to, if not identical with, softened fibrine.

3rd. That the softening of coagulated fibrine is an elementary pathological condition of frequent occurrence, distinct from suppuration, and constituting a considerable proportion of the cases generally denominated suppurative phlebitis.

And he winds up his interesting paper thus:—

"Lastly, I may observe, that I have frequently found pus mixed in considerable quantities with the pulpy fibrinous matter; and in two instances some purulent globules were observed, completely insulated, in the centre of a clot, from the neighbouring tissues. But in both these cases pus was also detected in the blood; and when it is considered how frequently this contamination exists, it will not appear surprising that the particles of pus should become entangled in coagula contained in the vessels. Nor does this circumstance at all invalidate the conclusion, that the softening of coagulated fibrine is a disease of much interest and importance, quite distinct in its nature from suppuration, although the two affections have been generally regarded as identical." 152.

We would simply remark that Mr. Gulliver's researches on the presence of pus in the blood are far from being established. There are grave doubts entertained of their correctness.

### XVII. Case of Carditis. By Thomas Salter, Esq. of Poole.

Mr. Salter points out the absence of any recorded case of pure carditis, unaccompanied with inflammation of the pericardium. He relates the following as a not unsatisfactory approach to it.

N. P. Sheppard, aged 50, March 16, 1834. He complained of uneasy sensations in the region of the stomach, and under the sternum, increased by any sort of exertion. The appetite was good, and the pain not influenced by eating; bowels regular; tongue clean at the edges, but the centre and back parts covered with a thin white coating; pulse natural; countenance pale. On applying the ear to the cardiac region nothing abnormal was discovered.

He states that the symptoms were first observed about six weeks since, whilst walking; that the pain was then at the lower part of the chest, inclining to the left side, and though it did not continue long was remarkably severe, producing faintness and a cold perspiration. About a week subsequent to this he had another severe paroxysm, which occurred immediately after a walk of three miles into the country. The attacks are becoming more frequent, though not always so severe as the first; they now occur occasionally whilst sitting, and even lifting the arm sometimes brings on the pain. Accompanying the pain in the chest there is frequently considerable uneasiness about the middle of the left upper arm. There is no cough or sign of any pulmonary affection. He complains a good deal of flatulence, and says he is made worse by the use of beer. He was directed to take some pills with pil. hydrar. and ext. colocynth every other night, and twice in the day a portion of a mixture containing sulphate of magnesia and carbonate of soda: from the use of these remedies, and a strict attention to regimen, he experienced considerable relief; till early in the morning on Saturday the 22nd, when Mr. S. was hastily called to him.

"I found him sitting up in his bed, being, from great oppression and distress in his breathing, unable to lie down. He placed his hand over the sternum, and said with great earnestness, 'it all lies here.' The pain was not lancinating, but of a dull heavy description; his face was pale, features sharp, and the expression anxious; and he said he felt as if he could not possibly live; pulse 80 and regular; heat of the skin temperate; pain in a slight degree increased by deep breathing; no tenderness at the scrobiculus cordis; feels some relief by raising wind from the stomach; action of the heart seems natural; chest sounds well on percussion, and the respiratory murmur is good.

He was bled to ten ounces, and had a blister applied over the sternum: in the evening there was no remission of the symptoms; his sufferings continuing distressingly severe, and the seat and character of the pain remained unchanged; the pulse had risen in frequency to 120, and was small and weak; on inspiring deeply the pain was but in a slight degree increased; pressure on the intercostal spaces did not produce pain. The respiratory murmur was heard on both sides of the chest, but the anguish of the patient was so great, that it would have been extremely difficult, if not impossible, to have made any very satisfactory local examinations. His sufferings and distress in breathing were at this time so great that he did not appear able to give any precise or intelligible account of his feelings. He was directed to take one grain of opium and four of calomel every three hours." 75.

23d. He has passed a sleepless night, with restlessness and anxiety almost indescribable, frequently getting in and out of bed, and once dressing himself. He has vomited a large quantity of fluid tinged with green bile. He is still constantly obliged to observe the erect position; pulse 130, and so feeble as scarcely to be felt; sounds of the heart just distinguishable by the stethoscope; surface of the body cool; bowels open; urine in moderate quantity, with sediment. The pain under the sternum is less complained of, but the oppression of the breathing and the anxiety are truly distressing to witness.

In the evening, he complained of pain in the upper and left side of the chest, and beneath the clavicle. He had no sleep during the night, and, on the 24th, the pulse was not to be felt at the wrist; the hands, feet, and legs are cold; the patient continued to suffer greatly during the day, but gradually becoming weaker, he died at eleven o'clock at night, about 65 hours after the accession of the acute symptoms. His mind was unaffected throughout.

*Dissection.*—In the right pleural cavity there was more than half a pint of serous fluid. Nearly the whole of the left lung was in a state of considerable engorgement, resembling the first stage of pneumonia; it was crepitant, but from the cut surfaces escaped a large quantity of sero-sanguineous fluid, and from one of the cut bronchial tubes a clear fluid was observed to flow, which by pressure was also forced out of the trachea. The capillary and other vessels on the bag of the pericardium were distended with red blood, especially on the left side, exhibiting a beautiful vascularity. That portion of the membrane investing the substance of the heart showed also, by its unusual vascularity, striking evidence of having suffered from inflammation; this, too, was mostly on the left side, that portion covering the left ventricle being its chief seat; but the part of the pericardium which had sustained the most intense vascular disturbance, was that which lies on, and is attached to, the diaphragm. Here there was not only a distended state of the capillary and larger vessels, but numerous ecchymosed spots and blotches resembling those observed on the skin in purpura hæmorrhagica. There was no lymphatic, serous, or purulent effusion into the pericardial sac. The heart was somewhat larger than natural, its substance of moderate firmness; large coagula were found in all the cavities; some of these were quite white, others deprived of their red colour, and of a yellowish hue, and adhering with great tenacity to the internal parietes, whilst some portions of the coagula were dark on the outside and bleached within. There were no signs of disease in the lining membrane of the heart or valves. In the ascending aorta there were the appearances of common ossification. The great deviation from the normal condition of the heart was, however, found to be in the muscular substance of the left ventricle; excepting a small portion of a few lines in thickness on either surface, the left ventricle had entirely lost its muscular colour; it was of a lightish yellow hue, but still preserving the fibrous character of muscle. From all the cut surfaces of the various sections which were made, could be scraped purulent matter. In some parts absorption had taken place, leaving small cavities in the muscular substance, varying from the size of a pin's head to that of a small pea; these were all filled with pus. The stomach was large and flaccid, and contained a considerable quantity of fluid. The mucous membrane of the pyloric half was of a dingy red colour, from a spotted vascularity. The liver appeared quite healthy. The jejunum was inflamed, both in its peritoneal and mucous coats, for the greater portion of its length.

The case speaks for itself.

### XVIII. ON THE STRUCTURE OF THE CORPUS LUTEUM. By ROBERT LEE, M.D. F.R.S.

Dr. Lee—

Haud sordidus auctor

Naturæ verique,—

Commences his paper by stating *that,* the Graafian vesicle in the human ovarium is a small spherical pellucid sac, which contains a fluid, the ovum, and the granular substance in which it is imbedded. The vesicle itself always consists of two distinct coats or membranous layers, which adhere firmly together. The external surface of the Graafian vesicle adheres loosely to the stroma or proper substance of the ovarium, in which it is imbedded by soft cellular substance, blood-vessels, and nerves.

Soon after impregnation, the coats of the Graafian vesicle and the peritoneum covering it, give way by absorption, the contents of the vesicle escape, and between its outer coat and the substance of the ovary, the corpus luteum is gradually formed.

After quoting the opinions of Baer and Montgomery on the site of the corpus luteum, Dr. Lee details his own researches.

On the 11th of July 1838, a woman, two months advanced in pregnancy, died of continued fever in St. George's hospital. On the 13th, Dr. Lee examined the left ovarium, which contained the corpus luteum.

"It was larger than the right ovarium, and had a considerable prominence on its convex edge, around which were seen ramifying a number of minute arteries and veins. There was a small circular depression at the point of this prominence, but a bristle could not be made to pass through it into the substance of the ovarium. On cutting open the ovarium, the corpus luteum presented itself of an oval shape and deep orange colour, with a small cyst in its centre, resembling the Graafian vesicle, with its coats thickened and contracted. With little difficulty I succeeded in separating one half of this cyst into two distinct layers which appeared to be the two coats of the Graafian vesicle.

The outer surface of this cyst is so loosely attached by cellular tissue to the corpus luteum, that it can easily be separated from it. The corpus luteum itself varies from a line to a line and a quarter in thickness, and when examined with a magnifier, appears to consist entirely of small yellow globules or particles contained in cellular membrane.

Around the outer surface of the corpus luteum, and completely investing it, there is a white layer varying in thickness, the outer part of which loses itself in the substance of the ovarium, of which it appears to form a part, and to be similar in structure, having the mouths of divided vessels distinctly perceptible, as in other parts of the substance of the ovarium. The inner portion of this white layer, which appears to be condensed stroma, is separable on the one hand from the corpus luteum, and on the other from the substance of the ovarium, so as to give the appearance of a distinct membrane, considerably exceeding in thickness both layers of the Graafian vesicle. Plate VII. fig. 1 exhibits the colour and form of the corpus luteum when first cut open, with the Graafian vesicle in its centre.

The Graafian vesicle is also enclosed within the corpus luteum, in a specimen of Fallopian tube conception of six or seven weeks, in my collection.

In another specimen of more advanced tubal conception, the Graafian vesicle is likewise seen enclosed within the corpus luteum.

The same fact is fully as evident in the preparation of the gravid uterus of ten weeks, in my paper on the membranes of the human ovum in the 17th volume of the Transactions of the Society; and in several of the preparations of the gravid uterus in the Hunterian Museum, the Graafian vesicle is also contained within the corpus luteum, and forms its central cavity." 333.

So Dr. Lee concludes that the corpus luteum is neither produced by a thickening of the inner layer of the Graafian vesicle, nor by a deposit of a new substance between its two coats, but that it is formed around the outer surface of both these coats of the Graafian vesicle, and that the stroma of the ovarium is in immediate contact with the external surface of the yellow matter.

As gestation advances, Dr. Lee continues, the deep yellow colour of the corpus luteum fades, and the Graafian vesicle in its centre contracts and assumes a peculiar white membranous appearance, with small bands passing outward through the substance of the yellow matter, like the radii of a circle.

The corpus luteum has almost completely disappeared, and the ovarium returned to its natural size about three months after parturition. A small depression on the surface, and a slender white line running into the substance of the ovarium, are all the traces of the corpus luteum which remain in an ovarium three months after delivery.

" In the ovaria of women who have never been pregnant, yellow oval-shaped bodies are frequently found, which it is difficult to distinguish from true corpora lutea.

In the greater number of spurious corpora lutea, as Dr. Montgomery has observed, the appearances are produced by blood extravasated within the Graafian vesicles, which assumes a fawn hue as the colouring matter disappears by absorption, and undergoes various changes, similar to those which are observed to take place in coagula of blood formed in the cavities of veins from inflammation of the coats or mechanical obstruction. After a longer or shorter period, the blood is entirely removed, and the coats of the vesicle contract, and often assume a brown, yellow, or black colour. In these false corpora lutea, the yellow matter is contained within the Graafian vesicle, and does not form around it, as true corpora lutea are always observed to do.

In advanced life a thickening of the layers of the Graafian vesicle not unfrequently gives rise to appearances resembling corpora lutea. These, and all other false corpora lutea, are generally found deeply imbedded in the substance of the ovarium, or, if they are near the surface, they are not actually in contact with the peritoneum, but have a small portion of stroma intervening. If there is a cicatrix over these, it has an irregular form, very unlike the small circular aperture always seen in the peritoneum covering the true corpus luteum. Besides, in true corpora lutea there are always bands running from the outer surface of the central capsule to the stroma, surrounding the yellow substance of the corpus luteum." 335.

Appearances which might be mistaken for true corpora lutea have been observed in the ovaria of women who have died during menstruation.

" On the 18th of November 1832, I examined the uterus and the ovaria of a young woman who had died suddenly the preceding day, when the catamenia were flowing. Both ovaria were larger than usual, and the Fallopian tubes were red and turgid. The peritoneal coat of the left ovarium was perforated at that extremity nearest the uterus by a small circular opening, around which the surface of the ovarium was elevated, and of a bright red colour. When cut into, the substance of the ovarium around had a fawn colour.

On the 14th of January 1837, a woman thirty-seven years of age, who had long suffered from hysteria, died suddenly in St. George's Hospital, during menstruation. No morbid appearance was found to account for her death. A small circular aperture was observed in the peritoneum of the left ovarium, near the point where the corpus fimbriatum is fixed to the extremity of the ovarium. This opening communicated with a cavity in the substance of the ovarium, which was surrounded with a soft yellow substance, of an oval shape. The distinctive characters of the true corpus luteum were wanting.

From all the observations hitherto made upon the true corpus luteum, we may conclude that it is never formed but as a consequence of impregnation. The yellow oval-shaped substances found in the ovaria of women who have never been pregnant, are produced by morbid states of the Graafian vesicles, and are essentially different in structure.

P.S. On the 27th of July 1839, a lady, 29 years of age, died in the second month of her first pregnancy; and I inspected the body on the 29th, with Mr. Jorden of Lower Belgrave-street. The right ovarium contained the corpus luteum, from which there escaped about a small tea-spoonful of yellow serous fluid when it was cut open. On the 30th of July, I examined the ovarium and corpus luteum with Sir Astley Cooper and Mr. Wharton Jones, and the result is, that the correctness of the view which has been taken of the structure of the corpus luteum in this paper is now put wholly out of doubt. From the preparation of the part and the fac simile made of it by Mr. Jones, it is evident that no capsule surrounds the yellow matter, but that the outer surface of the yellow matter is in immediate contact with the stroma, or proper tissue of the ovarium. It further clearly appears, that both the layers of the Graafian vesicle are within the yellow matter, that the innermost of these layers is smooth, and the outer layer rough and filamentous, and that processes are sent out from this exterior layer which penetrate the yellow matter to a considerable depth, and in some parts go quite through it to the stroma of the ovary. The peculiar convoluted appearance of the yellow matter is also distinctly seen." 337.

Nothing can seem more satisfactory than this communication of our able and excellent friend, Dr. Lee.

This concludes our account of the present volume of the Society's Transactions—Transactions which still maintain their high character, and are eminently worthy of the medical literature of the country.

